# First discovery of charcoal-based prehistoric cave art in Dordogne

**DOI:** 10.1038/s41598-023-47652-1

**Published:** 2023-12-14

**Authors:** Ina Reiche, Yvan Coquinot, Antoine Trosseau, Anne Maigret

**Affiliations:** 1 IRCP UMR 8247 & New AGLAE FR 3506 CNRS-C2RMF-ENSCP/PSL University, Centre de Recherche et de Restauration des Musées de France (C2RMF), 14 quai François Mitterrand, 75001 Paris, France; 2 Centre de recherche et de restauration des musées de France (C2RMF), 14 quai François Mitterrand, 75001 Paris, France; 3 Present Address: Université de Caen-Normandie, Caen, France

**Keywords:** Environmental sciences, Chemistry

## Abstract

Archaeologists have long been puzzled by the exact age of Paleolithic cave art in Europe especially in the Franco-Cantabrian region with hundreds of decorated caves because the creation of this parietal art (paintings, drawings and engravings) is closely tied to the appearance of first modern humans in Europe and their ways of life. The Dordogne region, one of the richest regions in terms of Paleolithic cave art in the world with more than 200 cave sites, is currently known to provide figures of cave art solely made with mineral coloring matters that cannot be dated directly. Using in-situ non-invasive Raman spectroscopy combined with portable X-ray fluorescence analysis as well as visible and infrared imaging of the decor of the Font-de-Gaume cave, we show the presence of a large number of charcoal-based Paleolithic figures besides others made of iron and manganese oxides in the main galleries for the first time. The creation periods of the cave art at Font-de-Gaume are mainly attributed to the Magdalenian period and probably more complicated constituted of at least two creation phases than commonly established as shown by the direct or partial superimposition of carbon-based and iron- and/or manganese-based figures. Our new results contribute to a better understanding of the organisation of the ornamentation and thus of the imaginary language of our Prehistoric ancestors. The discovery opens new research possibilities for re-reading of the complex panels and absolute radiocarbon dating.

## Introduction

A large number of Carbon (charcoal)-based black graphical entities was discovered by our team between the 27th and the 29th of February 2020 in the décor of the main galleries of the Font-de-Gaume cave, Dordogne, Southern France. The Dordogne is one of the world’s richest regions in terms of decorated Paleolithic caves such as the famous Lascaux cave^1,2^. The decoration recognised in 1901 by Capitan, Peyrony and Breuil was known to be created using different iron (Fe) and manganese (Mn) oxide-based coloring matters, thus preventing direct radiocarbon dating^3^. The discovery of C-based cave art is therefore not only crucial for the Font-de-Gaume cave but for the whole Dordogne region.

The décor of the Font-de-Gaume cave shows naturalist mono-, bi- and polychrome paintings as well as engravings of different animals and signs in their main galleries. A little over two hundred figurative depictions were counted and can be divided in approximately two thirds to animal art and one third to signs (tectiforms)^4,5^. Mainly bison (N 80), mammoths, deer, horses and two negative hands are represented, which is why Font-de-Gaume is also called “Bison Cave”^3^.  Although the identification and numbering of the figures present at Font-de-Gaume by Capitan et al.^3^ is lacking accuracy with respect to our current practices, it is still the only one published on the whole cave to our knowledge. Therefore, this numbering is adopted in this publication.

From a technical and a stylistic perspective, two types of animal representations can be distinguished. On the one hand, we have lifelike painted bison treated with bi- or polychromy, where painting and engraving are associated with the relief effects of the wall for an exceptional rendering. Different shades can be observed, ranging from black to brown and from red to yellow as well as the designs being mainly done in black and red. The archetype of “hyperbison”^6^ is found in many of these individuals. There are also drawn naturalist bison with black lines. On the other hand, there are graphical entities of different animals as well (bison, horse, deer) that are drawn and exhibit a more schematic style. Capitan et al. hypothesized at least two different styles of representations right after the discovery of the cave art in Font-de-Gaume^3^. Leroi-Gourhan also stated that at least two creation steps are noticeable from the outset in the Font de Gaume cave^7^. Evidence of different creation steps of the ornamentations have also been provided for the Bull Rotunda in the Lascaux cave and Pech-Merle, Lot, France^8–10^.

Henri Moissan, a French Nobel prize winner, identified in 1902 Fe and Mn oxides in the coloring matter samples from the Font-de-Gaume décor^11^. Today, this UNESCO world heritage site is protected and sampling is only allowed by authorities in highly exceptional cases. The awareness of the fragility of decorated cave sites has led to a transition from micro-sampling to non-invasive analyses, carried out in-situ^12–17^.

Scientific imaging was used to record figures and panels of the Font-de-Gaume cave. Areas of interest could be selected for in-situ point analyses by portable X-ray fluorescence analyses (pXRF) and micro-Raman spectroscopy. In-situ micro-Raman spectroscopy allows a precise identification of mineralogical phases of the Fe- and Mn-oxides and the detection of carbon (C)-based compounds^16,18,19^. In-situ pXRF enabled the determination of different types of Mn-oxide compounds in the black prehistoric figures^13,16^, even if this method presents intrinsic analytical difficulties linked to the strong penetration of X-rays^20^. Charcoal-black-based graphical entities could be identified and differentiated from other carbon (C)-based materials thanks to its characteristic broad scattering bands using Raman spectroscopy and deduced from the absence of a Mn signal in the in-situ pXRF spectra^18^. Moreover, false color infrared photography (FCIR) shows a different contrast for C-based figures with respect to the visible (VIS) image, which provides an extrapolation of the identified presence of C in small areas to parts or the whole figure. However, the observed systematic reddish contrast of C-based lines observed by FCIR in the graphical entities is not fully understood.

**Figure 1:**
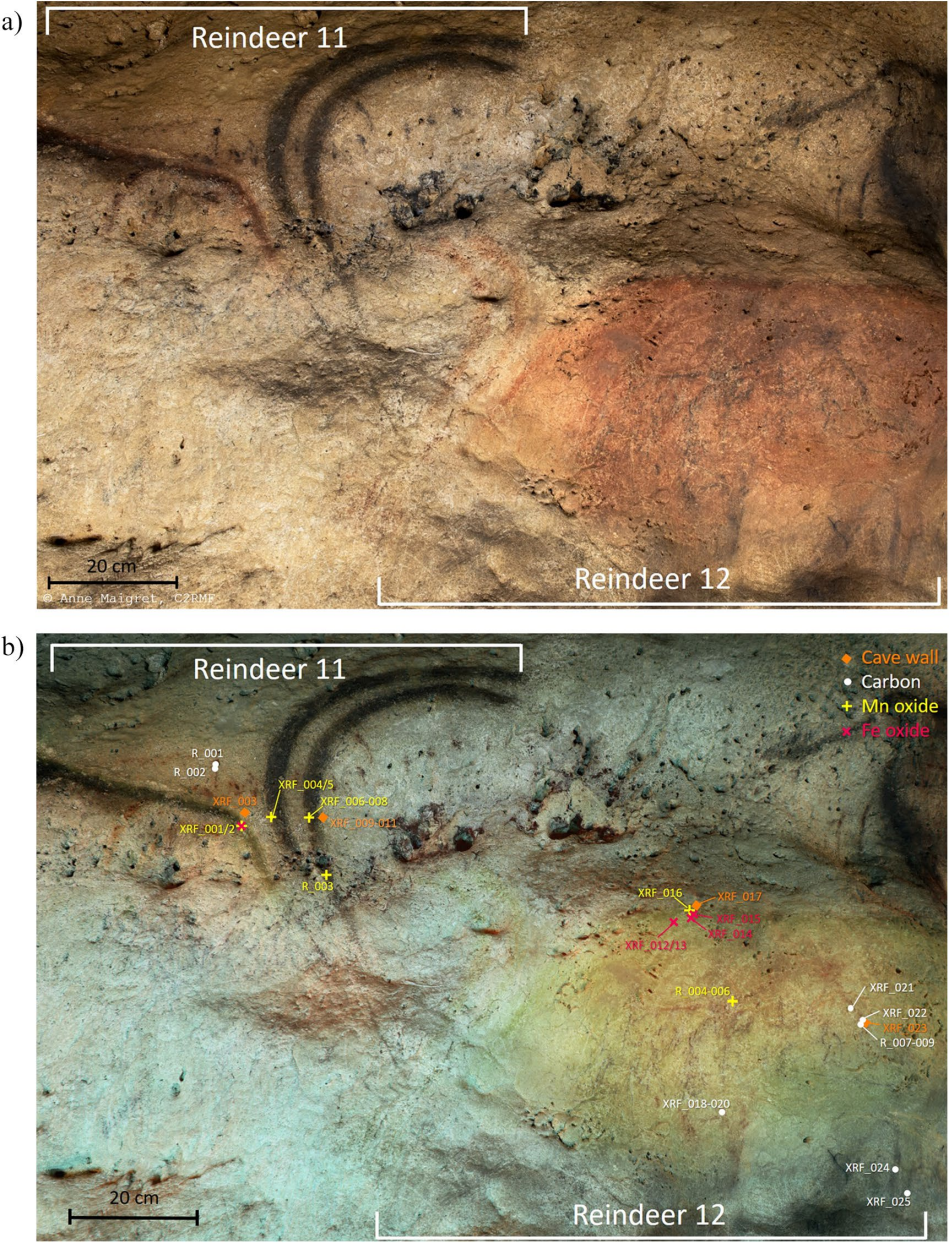
(**a**) Visible (VIS) light photography of the selected panel at the “Carrefour” ranging from the facing Reindeers 11 and 12 at Font-de-Gaume cave © Anne Maigret, C2RMF and (**b**) corresponding False colour infrared photography (FCIR) image of the facing reindeer evidencing the underlying black horse figure with the indicated analytical pXRF and micro-Raman point analyses.

**Figure 2:**
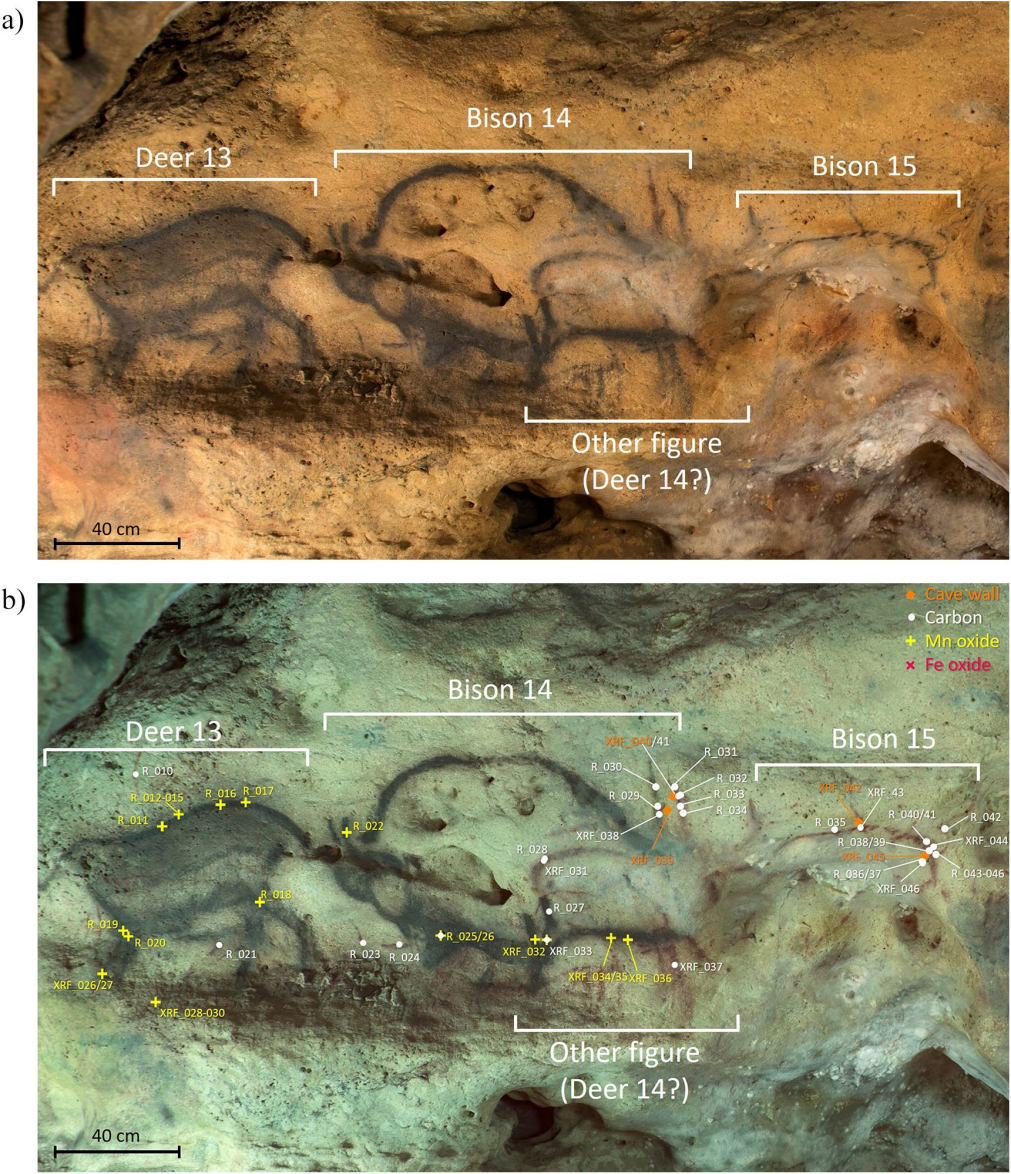
(**a**) VIS light photography of the selected panel at the “Carrefour” of the panels no. 13 and 14 composed of a reindeer and a horse as well as bison and possibly a deer, respectively, and the schematic Bison 15 © Anne Maigret, C2RMF and (**b**) corresponding FCIR image with the indicated analytical pXRF and micro-Raman point analyses.

The two types of naturalistic and schematic representations of animals are superimposed at the central crossing of the main and the lateral galleries of the Font-de-Gaume cave, the so-called “Carrefour”. This series of figure comprises the naturalistic facing Reindeers 11 and 12 (Fig. 1a), the naturalistic panel 13 with a reindeer likely superimposed with a horse that it difficult to read, the naturalistic panel no. 14 with a bison superimposed with another figure, possibly a deer, and the schematic Bison 15 in addition to some unidentified schematic marks (Fig. 2a). Above the Reindeer 11, there is a black dotted line, which crosses the animal representation below the black contour line. FCIR imaging revealed the presence of another animal below the Reindeer 12 that was not recognized until now, likely a black horse (Fig. 1b). Superimposed with the Deer 13, there are also unidentified black lines that appeared reddish in the FCIR image (Fig. 2b). A detailed description of the graphical entities, such as their artistic techniques, the in-situ measurement points and the analytical results, is given in the supplementary information (SI, Figure description).

**Figure 3:**
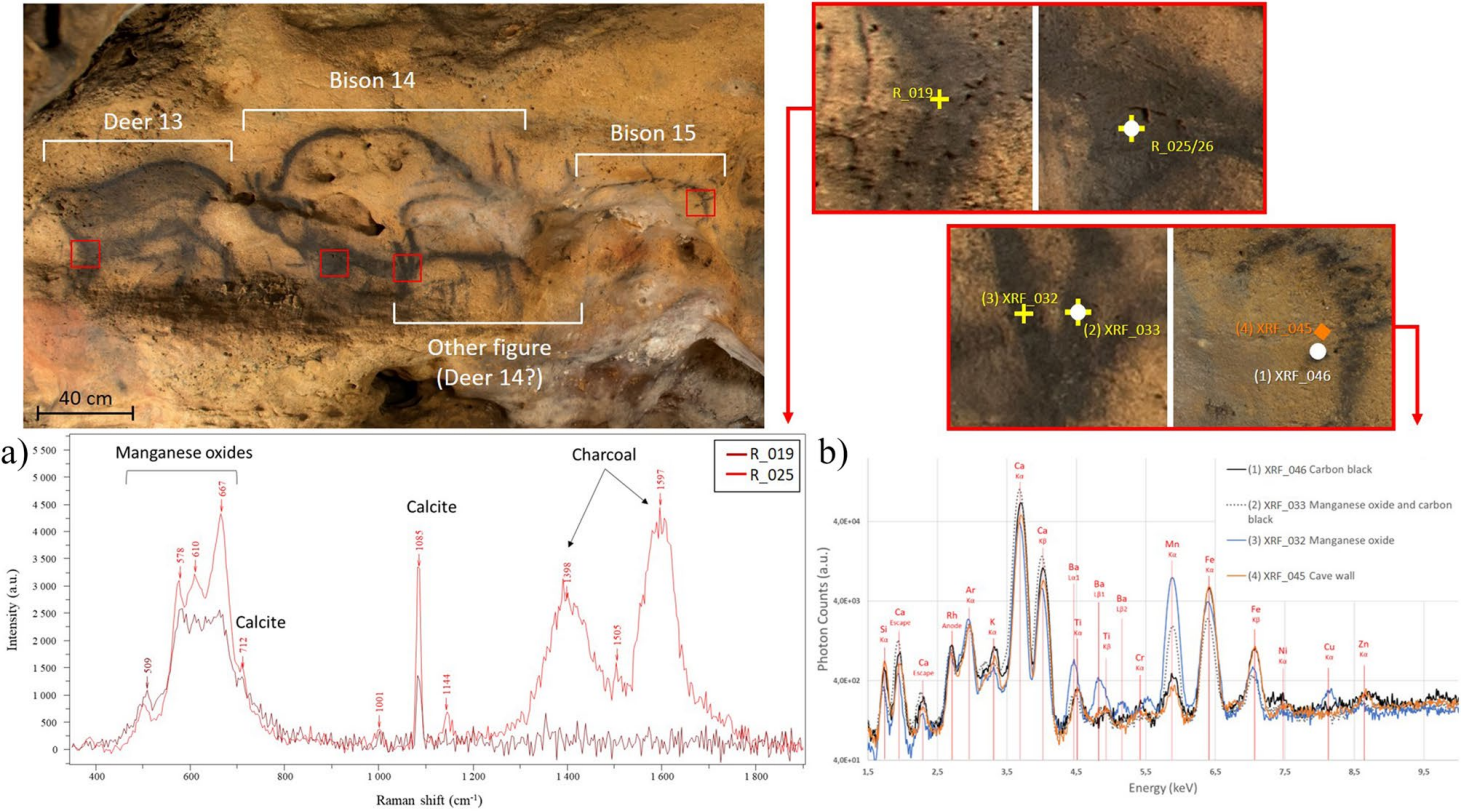
VIS light photography of the "Carrefour" with four analytical zones depicted in detail on the right-hand side: (**a**) in-situ Raman spectra of the Deer 13 (R019) and the Bison 14 (R025) with the analytical spots indicating the presence of charcoal, Mn oxides and calcite. The Raman bands around 1398 cm^−1^ and 1597 cm^−1^ are those of charcoal. The band at 1085 cm^−1^ is the main calcite band. Mn oxides are identified thanks to the bands between 500 and 670 cm^−1^, (**b**) in-situ pXRF spectra of the Bison 15, of the wall support and of the superimposed figures of the panel 14 showing different intensities of the Mn peak.

All black lines in the VIS image are not exhibiting the same contrast color in the FCIR image indicating the presence of different kinds of black coloring matter (Figs. 1b, 2b, SI Figure description). There are black lines in the VIS image that remain black in the FCIR images while others turn reddish after this image processing. In-situ pXRF showed that the black lines that appear black in the FCIR images correspond to Mn-based lines whereas those that appear reddish do not show a clear Mn-signal in their corresponding spectra (Fig. 3b, Table 1). These lines depict the typical C-based broad bands, especially with the shape of those of charcoal, at 1385 cm^−1^ and 1594 cm^−1^ in the Raman spectra (Fig. 3a), whereas the other black lines that remain black in the FCIR image show the presence of Ba bearing Mn oxide phases such as pyrolusite and romanechite (Fig. 3b). A semi-quantitative approach allowed the determination of the ratio of mineralogical phases in the Mn-oxide-based representations (Table 1, SI Table S1 and XRF data and spectra excel sheet) and thus a further discrimination between Mn–O-based figures. The chemical composition of the red areas of the Reindeers 11 and 12 is corresponding to Fe oxides, hematite, whereas the black lines of these figures correspond to the Mn oxides romanechite (0.6–0.8) and pyrolusite 0.2–0.4). The dotted line above the Reindeer 11 crossing the animal below is composed of charcoal as it shows the characteristic broad Raman scattering bands of charcoal and a reddish contrast in the FCIR image. The Deer 13 and the Bison 14 mainly show romanechite (Ba rich Mn oxide confirmed by pXRF) as coloring matter, whereas underlying marks and the underlying figure, possibly a Deer 14, show charcoal. The Bison 15 is entirely drawn with charcoal (Fig. 3a). This figure as well as the underlying figure 14 are stylistically more schematic in comparison to the other naturalistic bison and reindeers. Interestingly, these charcoal-based figures are displayed in the opposite direction as the above lying Mn–O-based reindeer and bison. A similar spectral response corresponding to charcoal is found for the unknown figure, likely the horse, below the red ventral area of the Reindeer 12. The head of this horse in charcoal is also directed in the opposite direction to that of the red Reindeer 12.

**Table 1:** Summary of the analytical results obtained on the different figures of the “Carrefour” at Font-de-Gaume cave.

XRF analysis	Raman analysis	Figure	Type of coloring matter (pXRF)	Type of coloring matter (Raman)	Color contrast in FCIR	Coloring matter inferred from FCIR
XRF_001–XRF_008	R_001–R_003	Reindeer 11	Fe oxide and mean Ba Mn oxide (group II^16^)	Mix of romanechite (Ba_2_ Mn_5_O_10_, xH_2_O)/pyrolusite (MnO_2_)	Black	Mn oxide
XRF_012–XRF_017	–	Backbone of Reindeer 12	Fe and Mn oxide	–	Black	Mn oxide
XRF_018–XRF_022 XRF_024–XRF_025	R_004–R_009	Black figure under Reindeer 12	Mn signal as low as in the wall support	Charcoal	Reddish	Charcoal
XRF_026/XRF_027	R_010–R_021	Panel 13 / Deer 13	Mn oxide	Charcoal	Black (because of super-imposition of charcoal with Mn oxide)	Mn oxide
XRF_028–XRF_030	–	Panel 13 / Black dots of an unidentified figure under Deer 13	Mn oxide	Charcoal	Reddish	Charcoal
XRF_032–XRF_036 XRF_037	R_023–R_027	Panel 14 / Bison 14 (+ black dots around it)	High Ba Mn oxide (group I^16^)	Charcoal	Reddish	Charcoal
				Mix of romanechite (Ba_2_ Mn_5_O_10_, xH_2_O)/pyrolusite (MnO_2_)	Black	Mn oxide
XRF_031 XRF_037 XRF_038–XRF_041	R_027–R_034	Panel 14 / other figure, possibly a Deer 14	Mn signal as low as in the wall support	Charcoal	Reddish	Charcoal
XRF_042–XRF_046	R_035–R_046	Bison 15	Mn signal as low as in the wall support	Charcoal	Reddish	Charcoal

Thus, we observe graphical entities in the galleries of the Font-de-Gaume cave that are made using charcoal that exhibit a schematic style below two types of Mn–O-based figures. The different types of coloring matter can be considered as distinctive characteristic of different creation phases according to observations in other comparable decorated caves such as Lascaux^2,8,10^ and can correspond to different imaginary languages of specific prehistoric groups. Our current knowledge indicates differences in the type of Mn–O-based coloring matter between the Lascaux (cryptomelane-based) and the Font-de-Gaume (romanechite and pyrolusite-based) caves but shows similarities between Font-de-Gaume and Rouffignac’s black coloring matter^16,21^. Above all, the identification of several charcoal-based figures below the characteristic Magdalenian style Mn–O-based and Fe–O-based figures opens new research avenues to chronologically classify the cave art more precisely by radiocarbon dating, improve the knowledge of creation phases, the frequentation of the cave site and, eventually, allow interregional comparison with absolute dates. The representation in a twisted perspective of the Bison 15, named "Picasso bison" by our team because of its schematic style, might be even more ancient than the Magdalenian period.

## Methods (including separate data and code availability statements)

### Photographic imaging

Photography under visible light (VIS) was performed with the use of two halogens on stand that allows to obtain images in high definition (Nikon, 24 MPixel) with well-lit figures for identification of the areas to be analyzed and the detailed study of the figures without traveling on site.

Photographic imaging allows observing coloring matters at the scale of the panels and figures in the cave. The points for chemical analyses by pXRF and for mineralogical analyses by portable micro-Raman spectroscopy can be judiciously chosen. This also limits the number of analyses required per figure and allows extrapolating the results of the physico-chemical analyses to the whole figure or at least the remaining visible part of it. The results of the analysis can therefore be read and discussed in the prehistoric context at the scale of a figure, a panel or even an area in the cave.

### Image treatment

The false color infrared photography image (FCIR) is a composite image obtained by superimposing two images representing the same view, one in the VIS and the second in IR range.

The IR image is produced using a sensitive camera from 400 to 1000 nm associated with a filter placed in front of the lens, which cuts the visible light and only allows radiation with a wavelength above 900 nm. A more or less contrasted grayscale image is obtained. Its interpretation can be difficult because it is depending on the properties of the pigments to absorb the IR light. In the colorimetric space, the red, green and blue (RGB) parts of the IR image are combined with the red and green layers of the VIS image using Photoshop^®^. The gray levels of the IR are then translated into color, and a new false color image (FCIR) is obtained. This association of both types of images makes it possible to differentiate different materials, which can appear very similar in the VIS image.

### Experimental conditions of portable X-ray fluorescence analysis (pXRF)

Portable XRF analyses were conducted in-situ in the cave with the portable ELIO device of the brand XG-Lab/Bruker^®^ of the C2RMF fixed on a tripod with two axial translations (forward and backwards as well as left to right) and three rotation axes. This standardized device holds a 50 kV X-ray tube with a Rh anode that can deliver a power of 4 W. The detector is a 17 mm^2^ SDD whit an energy resolution of 140 eV at the Mn Kα line. It is also equipped with a collimator able to focus the beam into a 1 mm spot, two lasers, a positioning system and a tripod. The distance between the measuring head and the wall is 5 mm. It also benefits of a camera for the observation of the measurement spot. The experimental conditions were 600 s of acquisition time with 40 kV and 40 µA delivered by the X-ray tube.

### Data treatment procedures

#### Linear regression based on elemental ratios

This protocol aims at bringing a first description of the black coloring matter based on Mn oxides by discriminating several groups corresponding to different phases or mixtures from spot measurements in pXRF directly on the prehistoric paintings. It has been previously used by other scholars^20^ but it was checked and improved for the current case study of the decor of the Font-de-Gaume cave^16^.

The pXRF spectra obtained during these measurements are processed with the PyMca software^22^. This software allows to fit the elemental peaks and to obtain an intensity in number of counts. As stated above, we use the intensity of Ba and Mn in this protocol. The signal of Mn is several orders of magnitude stronger in the coloring matter than in the substrate, while Ba is only found in the coloring matter. Thus, it is possible to use the signal ratios of these elements as distinction markers of the different types of coloring matters used. Depending on the Ba/Mn intensity ratio, several raw materials are then differentiated. In practice, the intensities of Ba are plotted versus those of Mn in a graph, and a linear regression is performed, allowing to assess the uncertainty. By doing so, an offset appears in the linear regression, so the meaningful quantity to study is actually the slope of these curves. Last but not least, this protocol allows studying coloring matters that are not pure because only two characteristic elements are used in the process.

### Experimental conditions of portable micro-Raman spectroscopy

The mobile Raman measuring device used was developed by Jobin–Yvon HORIBA and consists of components that can be easily moved during a measurement campaign. The device is equipped with a “Superhead SH 532” measuring head with an Olympus “long working distance” objective. In-situ Raman measurements require an arrangement with high flexibility and high stability at the same time. A working distance of 10 mm between the head and the wall guarantees the safety of the painted wall. The head is mounted on a stable tripod with one axial translation (forward and backwards) and three rotation axes. The measuring head is connected to an air-cooled Nd: YAG laser (Coherent DPSS 532; λ = 532 nm; laser power on the sample lower than 5 mW) and to the spectrometer via a 5 m long, flexible fiber optic cable. The Raman device is mounted with the HE 532 spectrometer from Jobin–Yvon HORIBA. A Peltier-cooled CCD detector (only for λ = 532 nm laser excitation; focal length 300 mm; fixed grating 920 lines/mm; fixed wavenumber from 100 to 3215 cm^−1^; spectral resolution of about 3 cm^−1^) was used for quick identification of the coloring matter. The integration time during the measurements was ranging between 1 and 6 s with 10 to 30 accumulations. Analogous conditions were employed as those used at the Rouffignac cave^19^.

### Data treatment procedure

The spectra are evaluated with the LabSpec software^®^ in order to obtain the characteristic Raman shift. Spectra are treated by Edge filter artefact and baseline corrections. The baseline is subtracted (use of the region of interest 250–800/1200 cm^−1^). Peak fitting is performed by fitting the Raman bands with Gaussian-Lorentzian sum (amplitude) functions.

**Figure 4:**
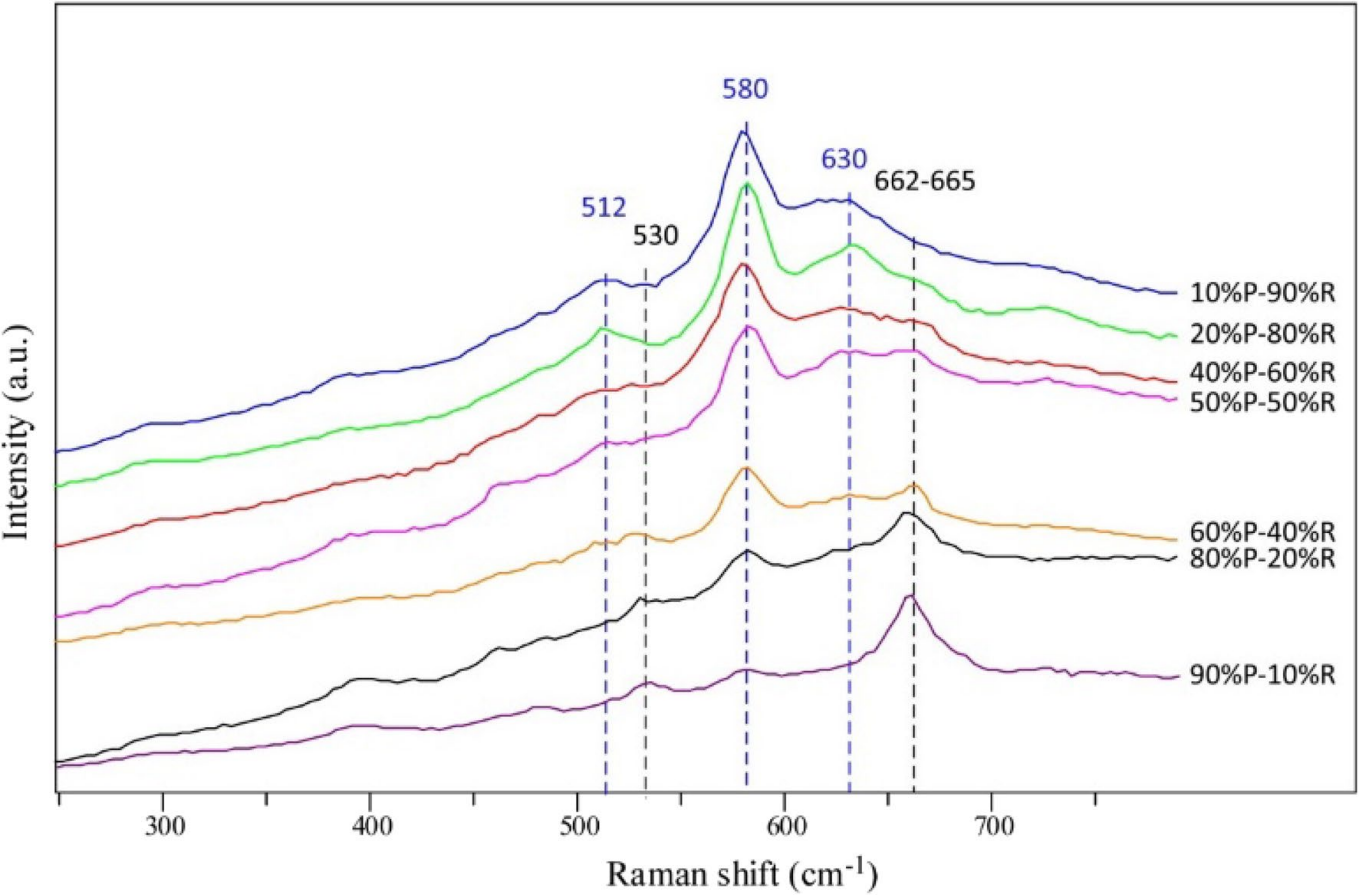
Raman spectra of different mixtures of geological pyrolusite (P) and romanechite (R) references measured with the same equipment, with their characteristic bands indicated.

Characteristic Raman bands of different mixtures of the geological references of the Mn oxides (pyrolusite and romanechite) are shown in the Fig. 4. The semi-quantitative evaluation of the Mn oxides is complicated because of the superimposition of different Mn oxide bands and the variation of the Raman signals with low intensity.

**Figure 5:**
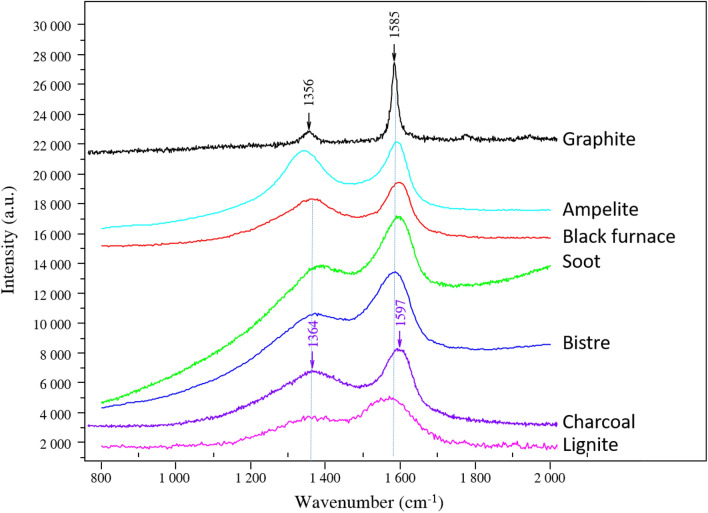
Raman spectra of different C-based references measured with the same equipment, with their characteristic bands indicated.

Different C-based references were studied as well^18^. The treated Raman spectra are represented in the Fig. 5.

### Ethics approval

Humans are not directly used in the study. We confirm that all methods were carried out in accordance with relevant guidelines and regulations. We confirm that all experimental protocols were approved by a named institutional and/or licensing committee. We confirm that informed consent was obtained from all subjects and/or their legal guardian(s).

## Data and code availability

All discussed data are either shown in the manuscript or in the supplementary information (SI). The codes used are either open source (PyMCA) or are acquired licenses by the C2RMF. The data are available through request to the corresponding author.

### Supplementary Information

Supplementary Information 1.

Supplementary Information 2.

Supplementary Information 3.
