# Supplementary material for: First discovery of charcoal-based prehistoric cave art in Dordogne
Source: Sci Rep. 2023 Dec 14;13:22235. doi: 10.1038/s41598-023-47652-1 (PMC10721606; doi:10.1038/s41598-023-47652-1)
Supplement: Supplementary file 1 — Supplementary Information 1. [file 41598_2023_47652_MOESM1_ESM.pdf]

Table S1 : Name of the XRF and Raman points of analysis

Raman point	Raman spectrum	XRF point	XRF spectrum
R_001	AP_FDG_2021_138_point_N_sur_renne11	XRF_001	AP_281119_13_FdG_Renne11_B
R_002	AP_FDG_2021_139_point_N_sur_renne11	XRF_002	AP_281119_14_FdG_Renne11_B
R_003	AP_FDG_2020_65_Renne11_noir	XRF_003	AC_281119_15_FdG_Renne11_P
R_004	AP_FDG_2021_136_renne12	XRF_004	AP_281119_05_FdG_Renne11_N
R_005	AP_FDG_2021_136b_renne12	XRF_005	AP_281119_06_FdG_Renne11_N
R_006	AP_FDG_2021_137_renne12	XRF_006	AP_281119_07_FdG_Renne11_N
R_007	AP_FDG_2021_133_renne12	XRF_007	AP_281119_08_FdG_Renne11_N
R_008	AP_FDG_2021_134_renne12	XRF_008	AP_281119_09_FdG_Renne11_N
R_009	AP_FDG_2021_135_renne12	XRF_009	AC_281119_10_FdG_Renne11_P
R_010	AP_FDG_2020_109_trait_noir_ss_renne13	XRF_010	AC_281119_11_FdG_Renne11_P
R_011	AP_FDG_2020_103_dos_renne13_noir	XRF_011	AC_281119_12_FdG_Renne11_P
R_012	AP_FDG_2020_102_dos_renne13_noir	XRF_012	AP_FDG_28112019_17_FdG_Renne12_R
R_013	AP_FDG_2020_101_dos_renne13_noir	XRF_013	AP_FDG_281119_16_FdG_Renne12_R
R_014	AP_FDG_2020_100_dos_renne13_noir	XRF_014	AP_FDG_28112019_18_FdG_Renne12_R
R_015	AP_FDG_2020_99_dos_renne13_noir	XRF_015	AP_FDG_28112019_19_FdG_Renne12_R
R_016	AP_FDG_2020_98_dos_renne13_noir	XRF_016	AP_FDG_28112019_20_FdG_Renne12_N
R_017	AP_FDG_2020_97_dos_renne13_noir	XRF_017	C_FDG_28112019_21_FdG_Renne12_P
R_018	AP_FDG_2020_66_Renne13_patte_arrG_noir	XRF_018	AP_FDG_281119_02_FdG_Renne12_PN
R_019	AP_FDG_2020_67_Renne13_poitrail_noir	XRF_019	AP_FDG_281119_03_FdG_Rennes12_N
R_020	AP_FDG_2020_68_Renne13_noir	XRF_020	AP_FDG_281119_04_FdG_Renne12_PN
R_021	AP_FDG_2020_108_trait_noir_ss_renne13	XRF_021	AP_FdG_C12_N_022021_23
R_022	AP_FDG_2020_105_corne_bison14_noir	XRF_022	AP_FdG_C12_N_022021_21
R_023	AP_FDG_2020_106_trait_vert_ss_tete_bison14_noir	XRF_023	C_FdG_C12_P_022021_22
R_024	AP_FDG_2020_107_trait_hori_ss_tete_bison14_noir	XRF_024	AP_FdG_R12_N_022021_20
R_025	AP_FDG_2020_110_machoire_inf_bison14_noir	XRF_025	AP_FdG_R12_N_022021_19
R_026	AP_FDG_2020_111_machoire_inf_bison14_noir	XRF_026	AP_FdG_C13_N_022021_30_test
R_027	AP_FDG_2020_95_arr_train_renne14_ou_pate_Bis14_noir	XRF_027	AP_FdG_C13_N_022021_31
R_028	AP_FDG_2020_96_dos_renne14_noir	XRF_028	FdG_FriseNoir_P08
R_029	AP_FDG_2020_90_corneG_renne14_noir	XRF_029	FdG_FriseNoir_P09
R_030	AP_FDG_2020_89_corneG_renne14_noir	XRF_030	FdG_FriseNoir_P10
R_031	AP_FDG_2020_91_corneD_renne14_noir	XRF_031	AP_FdG_29022020_16_Cerf14_N
R_032	AP_FDG_2020_92b_corneD_renne14_noir	XRF_032	AP_FdG_B14_N_022021_29
R_033	AP_FDG_2020_93_corneD_renne14_noir	XRF_033	AP_FdG_B14_N_022021_28
R_034	AP_FDG_2020_94_corneD_renne14_noir	XRF_034	AP_FdG_B14_N_022021_26
R_035	AP_FDG_2020_88_Bis_picasso_poils_dos_noir	XRF_035	AC_FdG_B14_P_022021_27
R_036	AP_FDG_2020_79_Bis_picasso_oeil_noir	XRF_036	AP_FdG_B14_N_022021_25
R_037	AP_FDG_2020_80_Bis_picasso_oeil_noir	XRF_037	AP_FdG_B14_N_022021_24
R_038	AP_FDG_2020_84_Bis_picasso_pt_antoine_noir	XRF_038	AP_FdG_29022020_14_Cerf14_RN
R_039	AP_FDG_2020_85_Bis_picasso_pt_antoine_noir	XRF_039	C_FdG_29022020_15_Cerf14_P
R_040	AP_FDG_2020_87_Bis_picasso_poils_tache_noir	XRF_040	AP_FdG_29022020_12__Cerf14_N
R_041	AP_FDG_2020_87b_Bis_picasso_poils_tache_noir	XRF_041	C_FdG_29022020_13_Cerf14_P
R_042	AP_FDG_2020_86_Bis_picasso_bout_corne_noir	XRF_042	C_FdG_29022020_11_Bison15_P
R_043	AP_FDG_2020_81_Bis_picasso_base_corne_noir	XRF_043	AP_FdG_29022020_10_Bison15_N
R_044	AP_FDG_2020_82_Bis_picasso_base_corne_noir	XRF_044	AP_FdG_28022020_08_Bison15_N
R_045	AP_FDG_2020_83_Bis_picasso_base_corne_noir	XRF_045	C_FdG_28022020_07_Bison15_P
R_046	AP_FDG_2020_83b_Bis_picasso_base_corne_noir	XRF_046	AP_FdG_28022020_06_Bison15_PN

Table S2: List of elemental peak intensities of XRF spectra acquired on figures (AP_XXX) or wall (C_XXX) for which Carbon is suspected due to absent or weak Mn signal (in bold) as well as for some adjacent Mn-traced figures, data 2019-2021. Yellow: Cave wall ; Green : Manganese oxide ; Blue : Charcoal ; Red : Iron oxide.

Figure	Point Sur Paroi	Al	Si	P	S	Ar	K	Ca	Ti	V	Cr	Mn	Fe	Ni	Cu	Zn	Rb	Sr	Y	Zr	Rh	Rh	Ba	Pb
Reindeer no. 11	C_FDG_28112019_10_FdG_Renne11_P	3,4E+02	1,1E+03	1,2E+04	7,4E+02	1,0E+04	2,6E+03	3,7E+05	7,0E+02	2,6E+02	4,5E+02	7,9E+03	1,6E+04	7,3E+02	3,9E+02	4,2E+04	N.D.	1,2E+03	7,3E+02	7,7E+02	9,8E+03	6,3E+03	N.D.	N.D.
	C_FDG_28112019_11_FdG_Renne11_P	3,3E+02	1,1E+03	1,2E+04	7,1E+02	9,1E+03	2,3E+03	3,3E+05	7,8E+02	N.D.	N.D.	7,1E+03	1,4E+04	6,6E+02	N.D.	3,7E+04	N.D.	9,0E+02	N.D.	N.D.	7,9E+03	7,3E+03	N.D.	N.D.
	C_FDG_28112019_12_FdG_Renne11_P	3,0E+02	9,3E+02	1,1E+04	7,0E+02	8,5E+03	1,9E+03	2,9E+05	6,6E+02	2,0E+02	4,5E+02	6,1E+03	1,2E+04	4,8E+02	N.D.	3,1E+04	N.D.	6,8E+02	N.D.	4,9E+02	3,0E+03	7,3E+03	N.D.	N.D.
	C_FDG_28112019_15_FdG_Renne11_P	2,8E+02	4,3E+03	7,2E+03	8,9E+02	9,1E+03	5,0E+03	2,6E+05	1,6E+03	9,0E+02	4,7E+02	1,2E+04	5,3E+04	6,9E+02	5,0E+02	2,1E+04	1,1E+03	1,2E+03	6,0E+02	6,1E+02	7,9E+03	7,7E+03	N.D.	N.D.
	C_FDG_28112019_21_FdG_Renne11_P	5,3E+02	5,4E+03	2,6E+03	7,6E+02	2,2E+04	1,1E+04	9,7E+05	3,1E+03	4,7E+02	7,4E+02	5,0E+03	6,1E+04	6,9E+02	N.D.	4,5E+03	9,5E+02	2,0E+03	8,8E+02	9,0E+02	1,5E+04	1,7E+04	N.D.	N.D.
	AP_FDG_281119_05_FdG_Renne11_N	3,7E+02	4,9E+03	5,2E+03	7,5E+02	1,6E+04	8,2E+03	2,2E+05	2,1E+03	9,3E+02	N.D.	1,6E+05	1,2E+05	1,2E+03	6,7E+02	1,4E+04	1,2E+03	1,9E+03	5,3E+02	1,0E+03	4,5E+03	1,4E+04	9,4E+03	2,0E+03
	AP_FDG_28112019_06_FdG_Renne11_N	2,0E+02	2,5E+03	2,8E+03	3,5E+02	8,8E+03	5,6E+03	1,3E+05	1,3E+03	4,7E+02	4,8E+02	8,4E+04	6,4E+04	9,0E+02	5,1E+02	8,5E+03	9,7E+02	1,3E+03	6,8E+02	8,4E+02	6,5E+03	6,8E+03	4,5E+03	1,3E+03
	AP_FDG_28112019_07_FdG_Renne11_N	3,0E+02	2,8E+03	4,4E+03	3,7E+02	1,0E+04	5,7E+03	2,1E+05	2,5E+03	1,8E+03	7,1E+02	2,9E+05	1,3E+05	1,4E+03	7,3E+02	1,4E+04	1,0E+03	2,8E+03	7,7E+02	1,9E+03	9,6E+03	7,4E+03	2,0E+04	2,2E+03
	AP_FDG_28112019_08_FdG_Renne11_N	3,4E+02	3,0E+03	4,7E+03	4,3E+02	9,3E+03	5,2E+03	1,9E+05	2,2E+03	1,8E+03	6,3E+02	2,7E+05	1,2E+05	1,2E+03	5,5E+02	1,3E+04	1,1E+03	2,5E+03	7,1E+02	1,7E+03	7,7E+03	8,3E+03	1,9E+04	2,0E+03
	AP_FDG_28112019_09_FdG_Renne11_N	3,1E+02	3,0E+03	4,7E+03	4,6E+02	8,4E+03	4,6E+03	1,7E+05	2,0E+03	1,5E+03	6,6E+02	2,4E+05	1,1E+05	9,2E+02	4,1E+02	1,1E+04	8,2E+02	1,8E+03	5,1E+02	1,0E+03	3,1E+03	9,0E+03	1,7E+04	1,7E+03
Backbone of Reindeer no. 12	**AP_FDG_281119_02_FdG_Renne12_PN**	2,8E+02	2,6E+03	5,2E+02	4,9E+02	9,8E+03	6,0E+03	3,4E+05	1,9E+03	1,4E+02	3,3E+02	**2,1E+03**	4,8E+04	3,1E+02	N.D.	1,1E+03	N.D.	1,0E+03	N.D.	N.D.	2,7E+03	8,7E+03	N.D.	N.D.
	**AP_FDG_281119_03_FdG_Rennes12_N**	2,4E+02	2,9E+03	4,1E+02	6,4E+02	9,1E+03	5,0E+03	3,2E+05	1,8E+03	1,2E+02	4,3E+02	**2,7E+03**	5,0E+04	3,7E+02	5,1E+02	1,1E+03	N.D.	6,9E+02	N.D.	N.D.	2,9E+03	8,7E+03	N.D.	N.D.
	**AP_FDG_281119_04_FdG_Renne12_PN**	2,9E+02	3,8E+03	7,0E+02	5,7E+02	9,2E+03	8,4E+03	3,3E+05	2,4E+03	3,0E+02	3,9E+02	**2,6E+03**	5,1E+04	6,2E+02	3,9E+02	1,4E+03	N.D.	9,0E+02	N.D.	6,3E+02	2,9E+03	8,9E+03	N.D.	N.D.
	**AP_FDG_28112019_20_FdG_Renne12_N**	5,4E+02	4,4E+03	3,9E+03	1,1E+03	2,1E+04	1,2E+04	6,8E+05	5,0E+03	5,9E+02	1,2E+03	**5,4E+03**	1,3E+05	1,3E+03	1,2E+03	1,0E+04	1,6E+03	2,8E+03	8,2E+02	1,6E+03	1,3E+04	1,6E+04	N.D.	2,8E+03
	C_FDG_28112019_21_FdG_Renne12_P	5,3E+02	5,4E+03	2,6E+03	7,6E+02	2,2E+04	1,1E+04	9,7E+05	3,1E+03	4,7E+02	7,4E+02	5,0E+03	6,1E+04	6,9E+02	N.D.	4,5E+03	N.D.	9,5E+02	2,0E+03	8,8E+02	9,0E+02	1,5E+04	1,7E+04	N.D.
Red pigment of Reindeer no. 12	AP_FDG_271119_05_FdG_Renne12_R	1,9E+02	4,2E+03	3,5E+02	1,5E+02	7,1E+03	3,9E+03	2,5E+05	3,8E+03	6,8E+02	N.D.	1,6E+03	3,6E+04	5,5E+02	2,9E+02	1,3E+03	N.D.	N.D.	N.D.	N.D.	N.D.	2,4E+03	6,4E+03	N.D.
	AP_FDG_281119_16_FdG_Renne12_R	5,3E+02	6,8E+03	3,5E+02	3,0E+02	9,2E+03	5,8E+03	1,6E+05	7,8E+03	1,2E+03	1,1E+03	2,5E+03	1,9E+05	1,0E+03	N.D.	2,4E+03	9,9E+02	1,0E+03	1,1E+03	6,1E+02	3,2E+03	7,2E+03	7,9E+03	N.D.
	AP_FDG_28112019_17_FdG_Renne12_R	9,8E+02	1,1E+04	3,8E+02	3,5E+02	1,8E+04	1,4E+04	5,7E+05	1,5E+04	8,8E+02	1,4E+03	4,7E+03	2,5E+05	1,3E+03	N.D.	3,3E+03	1,2E+03	N.D.	1,7E+03	N.D.	3,8E+03	1,5E+04	1,7E+04	N.D.
	AP_FDG_28112019_18_FdG_Renne12_R	7,2E+02	8,0E+03	2,6E+03	7,5E+02	2,0E+04	1,1E+04	6,4E+05	9,1E+03	9,8E+02	1,4E+03	2,3E+04	4,6E+05	9,2E+02	1,0E+03	5,9E+03	N.D.	9,9E+02	1,7E+03	1,3E+03	2,3E+03	1,5E+04	1,7E+04	N.D.
	AP_FdG_28112019_19_FdG_Renne12_R	5,5E+02	9,0E+03	2,9E+03	7,0E+02	1,8E+04	1,0E+04	5,4E+05	6,3E+03	1,3E+03	1,4E+03	1,6E+04	4,0E+05	1,5E+03	8,7E+02	7,6E+03	1,8E+03	1,7E+03	2,2E+03	1,3E+03	2,1E+03	1,6E+04	1,6E+04	N.D.
Black figure under Reindeer 12	**AP_FdG_R12_N_022021_19**	4,2E+02	6,2E+03	1,2E+03	6,1E+02	2,0E+04	1,1E+04	5,8E+05	5,0E+03	1,0E+03	7,9E+02	**8,0E+03**	1,2E+05	2,2E+03	9,8E+02	3,3E+03	7,4E+02	1,7E+03	1,2E+03	1,3E+03	1,5E+04	1,4E+04	N.D.	3,0E+03
	**AP_FdG_R12_N_022021_20**	4,7E+02	8,4E+03	1,1E+03	7,1E+02	1,9E+04	1,2E+04	4,4E+05	4,8E+03	1,1E+03	6,9E+02	**7,1E+03**	1,4E+05	1,2E+03	8,9E+02	3,1E+03	1,4E+03	1,9E+03	1,3E+03	6,6E+03	1,4E+04	1,6E+04	3,1E+03	N.D.
	**AP_FdG_C12_N_022021_21**	5,9E+02	5,2E+03	1,8E+02	5,6E+02	2,0E+04	1,0E+04	8,4E+05	1,3E+04	1,9E+02	7,3E+02	**7,8E+03**	8,7E+04	1,2E+03	1,2E+03	2,7E+03	N.D.	1,8E+03	1,4E+03	1,5E+03	1,4E+04	1,7E+04	1,2E+03	N.D.
	C_FdG_C12_P_022021_22	3,3E+02	8,9E+03	2,9E+02	5,0E+02	2,0E+04	8,9E+03	6,0E+05	2,9E+03	5,2E+02	8,4E+02	4,7E+03	7,2E+04	1,3E+03	9,2E+02	2,4E+03	N.D.	1,9E+03	N.D.	1,3E+03	1,4E+04	1,5E+04	N.D.	N.D.
	**AP_FdG_C12_N_022021_23**	5,6E+02	5,9E+03	4,3E+02	7,1E+02	2,0E+04	1,2E+04	7,2E+05	4,8E+03	3,2E+02	9,0E+02	**6,3E+03**	1,3E+05	1,6E+03	1,2E+03	3,3E+03	1,5E+03	2,3E+03	1,4E+03	1,6E+03	1,5E+04	1,6E+04	1,6E+03	N.D.
Deer no. 13	AP_FdG_C13_N_022021_31	3,2E+02	3,9E+03	1,3E+03	5,5E+02	2,4E+04	9,4E+03	4,5E+05	3,7E+03	9,4E+01	8,8E+02	2,5E+04	8,7E+04	1,2E+03	1,9E+03	5,3E+03	1,5E+03	1,9E+03	7,2E+02	1,1E+03	1,1E+04	1,6E+04	3,4E+03	3,2E+03
Black dots of an unidentified figure under deer no. 13	**FdG_FriseNoir_P08**	2,8E+01	2,7E+02	5,3E+02	3,8E+01	3,0E+03	9,3E+02	5,3E+04	4,0E+02	9,3E+01	1,7E+02	**2,5E+03**	1,2E+04	2,6E+02	5,5E+02	2,1E+03	4,7E+02	5,8E+02	N.D.	2,1E+03	2,4E+03	2,0E+03	3,3E+02	N.D.
	**FdG_FriseNoir_P09**	5,0E+01	6,8E+02	3,9E+02	6,3E+01	2,6E+03	1,7E+03	4,8E+04	1,2E+03	1,1E+02	2,5E+02	**2,6E+03**	4,2E+04	3,5E+02	3,0E+02	2,4E+03	5,9E+02	6,8E+02	N.D.	N.D.	2,6E+03	2,1E+03	N.D.	N.D.
	**FdG_FriseNoir_P10**	2,3E+01	5,7E+02	1,1E+03	8,8E+01	2,7E+03	1,7E+03	9,0E+04	7,9E+02	8,2E+01	2,1E+02	**2,5E+03**	1,6E+04	2,2E+02	3,1E+02	3,6E+03	3,8E+02	5,1E+02	4,2E+02	3,9E+02	3,0E+03	2,1E+03	N.D.	N.D.
	**FdG_FriseNoir_W11**	3,2E+01	6,3E+02	7,7E+02	6,9E+01	2,7E+03	1,9E+03	7,4E+04	6,9E+02	3,4E+02	1,4E+02	**2,5E+03**	1,9E+04	2,6E+02	2,3E+02	3,5E+03	N.D.	N.D.	4,3E+02	N.D.	2,4E+03	2,0E+03	N.D.	N.D.
Deer 14	**AP_FdG_29022020_12__Cerf14_N**	4,7E+02	1,3E+04	1,3E+03	6,2E+02	1,9E+04	1,0E+04	6,0E+05	3,4E+03	1,3E+03	4,6E+02	**9,6E+03**	8,8E+04	1,5E+03	7,5E+02	2,0E+03	1,2E+03	1,7E+03	N.D.	2,7E+03	1,5E+04	1,5E+04	N.D.	N.D.
	C_FdG_29022020_13_Cerf14_P	4,3E+02	4,1E+03	7,8E+02	3,5E+02	2,4E+04	9,1E+03	1,0E+06	2,2E+03	1,9E+02	4,4E+02	4,6E+03	4,8E+04	8,6E+02	7,0E+02	2,3E+03	N.D.	1,8E+03	N.D.	N.D.	1,3E+04	1,6E+04	N.D.	2,2E+03
	AP_FdG_29022020_14_Cerf14_RN	6,4E+02	4,6E+03	2,6E+02	6,0E+02	2,1E+04	1,2E+04	9,9E+05	4,1E+03	3,2E+02	5,5E+02	7,5E+04	2,4E+05	1,4E+03	5,2E+02	2,1E+03	1,2E+03	1,8E+03	N.D.	1,7E+03	1,5E+04	1,6E+04	5,3E+03	2,3E+03
	C_FdG_29022020_15_Cerf14_P	6,0E+02	6,0E+03	2,3E+02	4,7E+02	2,3E+04	1,5E+04	1,2E+06	3,6E+03	9,4E+01	7,4E+02	4,8E+03	5,4E+04	7,8E+02	5,5E+02	2,2E+03	1,5E+03	1,6E+03	N.D.	1,2E+03	1,6E+04	1,8E+04	N.D.	2,5E+03
	**AP_FdG_29022020_16_Cerf14_N**	3,3E+02	3,4E+03	1,5E+03	8,4E+02	1,9E+04	1,1E+04	4,5E+05	2,7E+03	6,4E+02	8,8E+02	**1,1E+04**	8,7E+04	1,1E+03	9,9E+02	1,9E+03	2,0E+03	1,8E+03	N.D.	1,3E+03	1,4E+04	1,7E+04	2,7E+03	2,5E+03

Bison 14	C_FdG_B14_P_022021_27	4,5E+02	4,3E+03	3,2E+02	5,1E+02	2,0E+04	9,4E+03	6,3E+05	4,9E+03	5,3E+02	6,7E+02	1,8E+04	9,6E+04	9,6E+02	6,6E+02	1,9E+03	1,4E+03	1,6E+03	N.D.	1,4E+03	1,3E+04	1,5E+04	N.D.	N.D.
	AP_FdG_B14_N_022021_24	1,1E+02	1,0E+03	5,7E+00	1,8E+02	1,2E+04	3,1E+03	1,5E+05	1,2E+03	1,2E+02	6,5E+02	4,1E+03	2,9E+04	3,7E+02	8,0E+02	8,3E+02	N.D.	6,9E+02	6,8E+02	N.D.	3,9E+03	7,5E+03	7,4E+02	N.D.
	AP_FdG_B14_N_022021_25	4,4E+02	8,9E+03	6,9E+02	6,4E+02	1,9E+04	1,1E+04	5,4E+05	3,5E+03	4,6E+02	1,5E+03	7,7E+04	1,0E+05	1,3E+03	1,2E+03	3,5E+03	1,3E+03	1,8E+03	9,4E+02	1,4E+03	1,4E+04	1,8E+04	1,1E+04	2,5E+03
	AP_FdG_B14_N_022021_26	5,5E+02	3,2E+03	4,4E+02	6,0E+02	2,0E+04	1,0E+04	7,4E+05	2,7E+03	1,1E+03	4,6E+02	2,1E+05	1,0E+05	7,3E+02	5,5E+02	2,4E+03	9,6E+02	2,4E+03	1,0E+03	1,4E+03	1,4E+04	1,6E+04	2,4E+04	2,8E+03
	AP_FdG_B14_N_022021_28	6,1E+02	2,1E+03	1,7E+02	5,6E+02	2,3E+04	8,2E+03	1,2E+06	1,4E+03	2,9E+02	4,6E+02	2,6E+04	3,2E+04	5,0E+02	6,9E+02	1,7E+03	N.D.	1,0E+03	N.D.	9,8E+02	1,4E+04	1,6E+04	2,8E+03	N.D.
	C_FdG_B14_P_022021_27	4,5E+02	4,3E+03	3,2E+02	5,1E+02	2,0E+04	9,4E+03	6,3E+05	4,9E+03	5,3E+02	6,7E+02	1,8E+04	9,6E+04	9,6E+02	6,6E+02	1,9E+03	1,4E+03	1,6E+03	N.D.	1,4E+03	1,3E+04	1,5E+04	N.D.	N.D.
	AP_FdG_B14_N_022021_29	2,3E+02	2,4E+03	1,2E+01	4,4E+02	2,5E+04	6,0E+03	4,5E+05	1,7E+03	5,6E+02	6,9E+02	1,1E+05	4,8E+04	5,8E+02	1,6E+03	1,2E+03	N.D.	1,6E+03	N.D.	1,1E+03	8,8E+03	1,6E+04	1,2E+04	N.D.
Bison 15	AP_FdG_28022020_06_Bison15_PN	2,3E+02	2,1E+03	1,1E+02	2,8E+02	1,1E+04	6,5E+03	4,3E+05	1,6E+03	2,0E+02	6,4E+02	2,7E+03	4,4E+04	9,7E+02	5,0E+02	1,2E+03	8,9E+02	1,0E+03	N.D.	N.D.	7,5E+03	9,4E+03	N.D.	1,5E+03
	C_FdG_28022020_07_Bison15_P	3,9E+02	5,2E+03	1,2E+03	5,6E+02	2,1E+04	9,0E+03	5,6E+05	3,3E+03	7,5E+02	8,8E+02	3,3E+03	8,6E+04	8,6E+02	N.D.	2,3E+03	1,2E+03	1,3E+03	6,1E+02	2,4E+03	1,3E+04	1,4E+04	N.D.	2,5E+03
	AP_FdG_28022020_08_Bison15_N	2,1E+02	3,4E+03	1,4E+03	4,3E+02	1,8E+04	5,1E+03	2,3E+05	1,8E+03	2,6E+02	6,3E+02	2,7E+03	5,6E+04	7,4E+02	8,9E+02	1,2E+03	8,3E+02	1,4E+03	7,0E+02	9,8E+02	9,5E+03	1,2E+04	N.D.	1,9E+03
	AP_FdG_29022020_10_Bison15_N	4,7E+02	3,6E+03	6,6E+03	7,5E+02	2,1E+04	7,8E+03	6,9E+05	2,3E+03	2,0E+02	9,0E+02	2,5E+03	5,0E+04	1,1E+03	9,0E+02	2,3E+03	6,2E+02	1,8E+03	7,9E+02	N.D.	1,5E+04	1,6E+04	N.D.	N.D.
	C_FdG_28022020_07_Bison15_P	3,9E+02	5,2E+03	1,2E+03	5,6E+02	2,1E+04	9,0E+03	5,6E+05	3,3E+03	7,5E+02	8,8E+02	3,3E+03	8,6E+04	8,6E+02	N.D.	2,3E+03	1,2E+03	1,3E+03	6,1E+02	2,4E+03	1,3E+04	1,4E+04	N.D.	2,5E+03
