# Supplementary material for: First discovery of charcoal-based prehistoric cave art in Dordogne
Source: Sci Rep. 2023 Dec 14;13:22235. doi: 10.1038/s41598-023-47652-1 (PMC10721606; doi:10.1038/s41598-023-47652-1)
Supplement: Supplementary file 3 — Supplementary Information 3. [file 41598_2023_47652_MOESM3_ESM.pdf]

Supplementary information for the manuscript:

First discovery of charcoal-based prehistoric cave art in Dordogne

by Ina Reiche[1,2*], Yvan Coquinot[3], Antoine Trosseau[1, 4], Anne Maigret[3]
1 PSL, Chimie Paristech, IRCP UMR 8247 CNRS - Centre de recherche et de restauration des musées de France (C2RMF), 11 rue Pierre et Marie Curie, 75005 Paris, France
2 FR 3506 New AGLAE CNRS-C2RMF, Louvre Museum, 14 quai François Mitterrand, 75001 Paris, France
3 C2RMF, Louvre Museum, 14 quai François Mitterrand, 75001 Paris, France
4 present address: University of Caen, France

Figure description. pXRF and Raman data with interpretation

Analysis point localizations

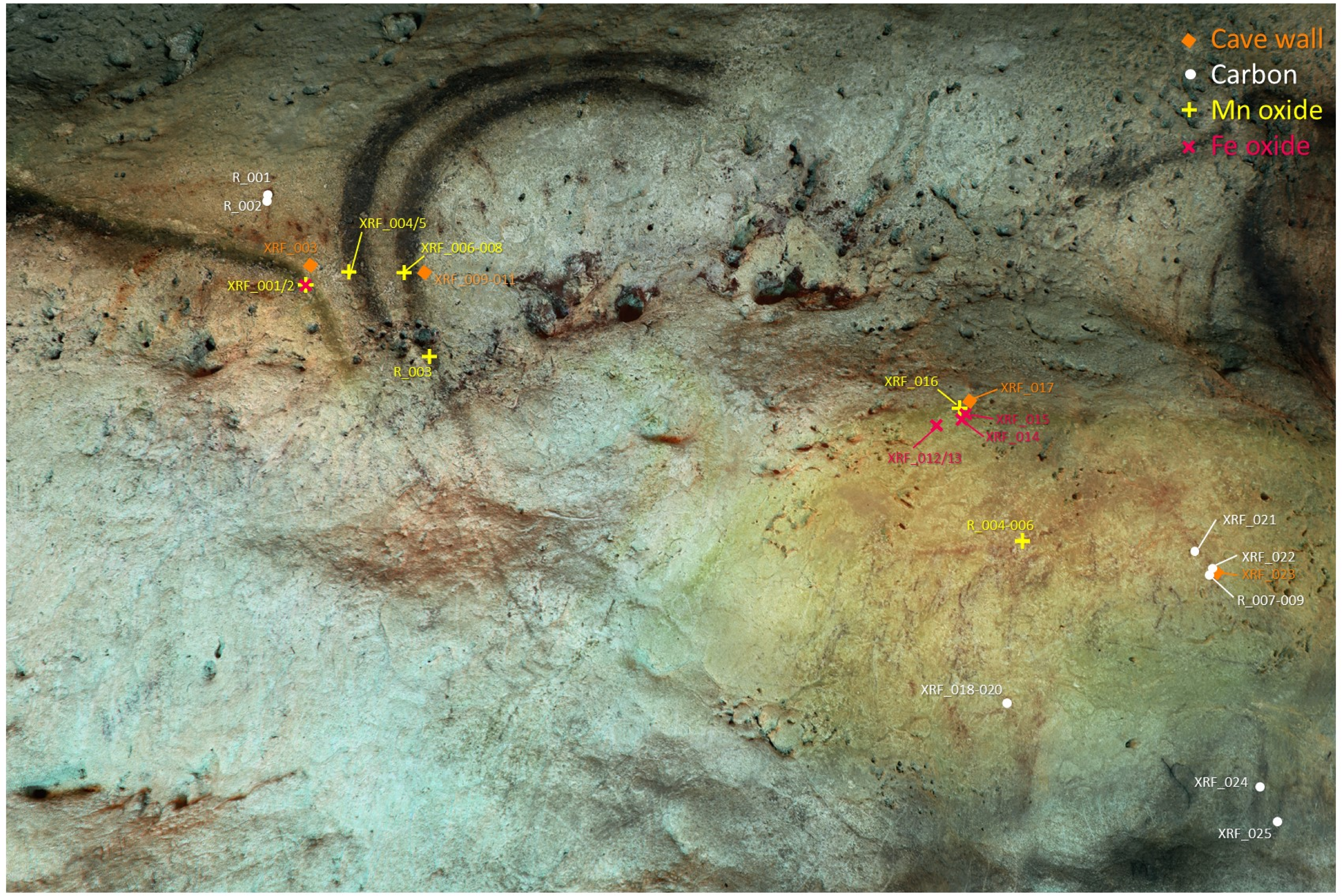

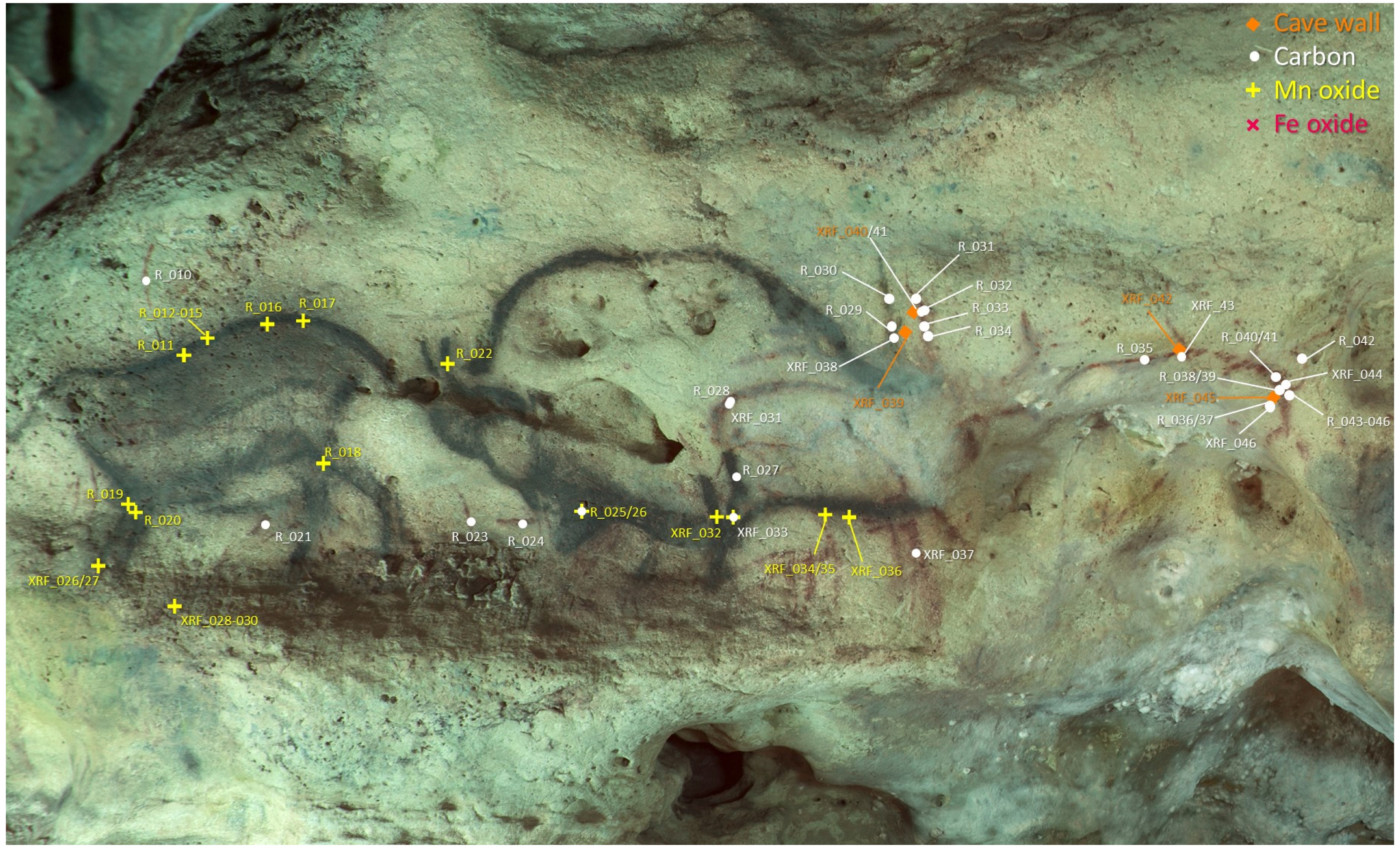

Reindeer no. 11

No superimposition
Painted and graved. Bichromic (black and red). Deeply engraved details. Outlines drawn in red and graved. Overall paint in black with gradient. Use of the wall relief.
Represented face to another reindeer of the figure 12 and Licks its muzzle. Directed to the right.
Partially faded.

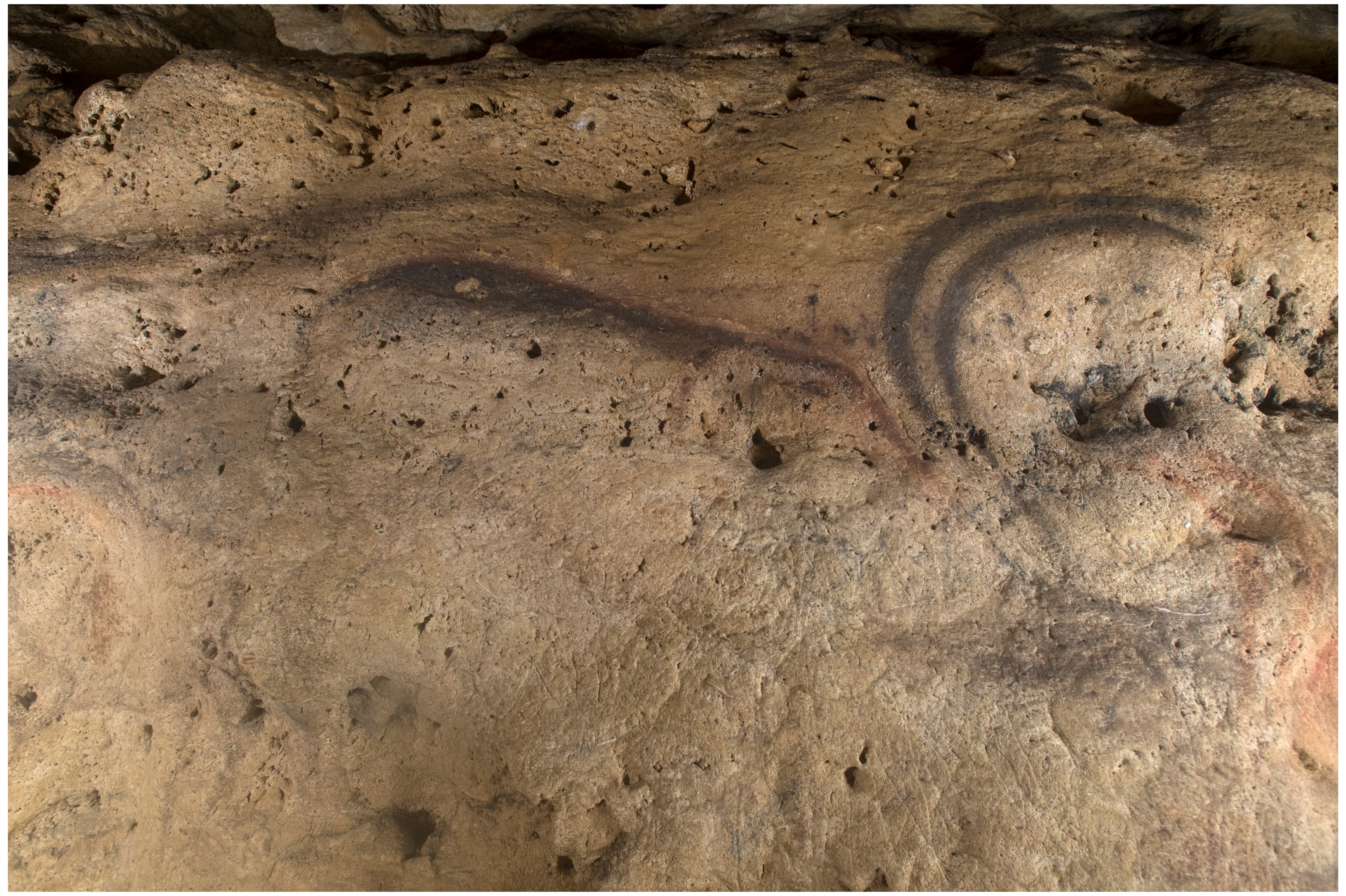

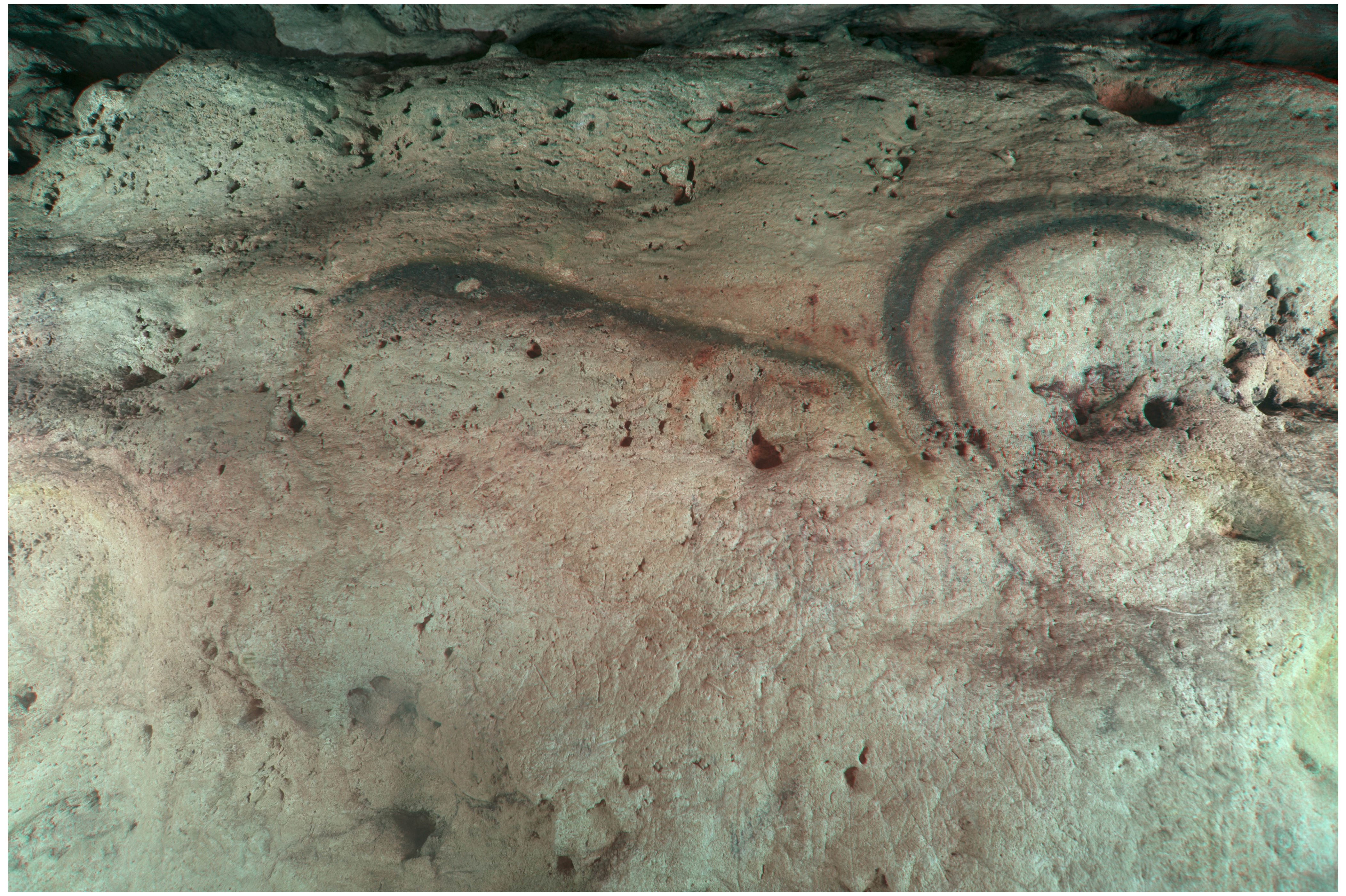

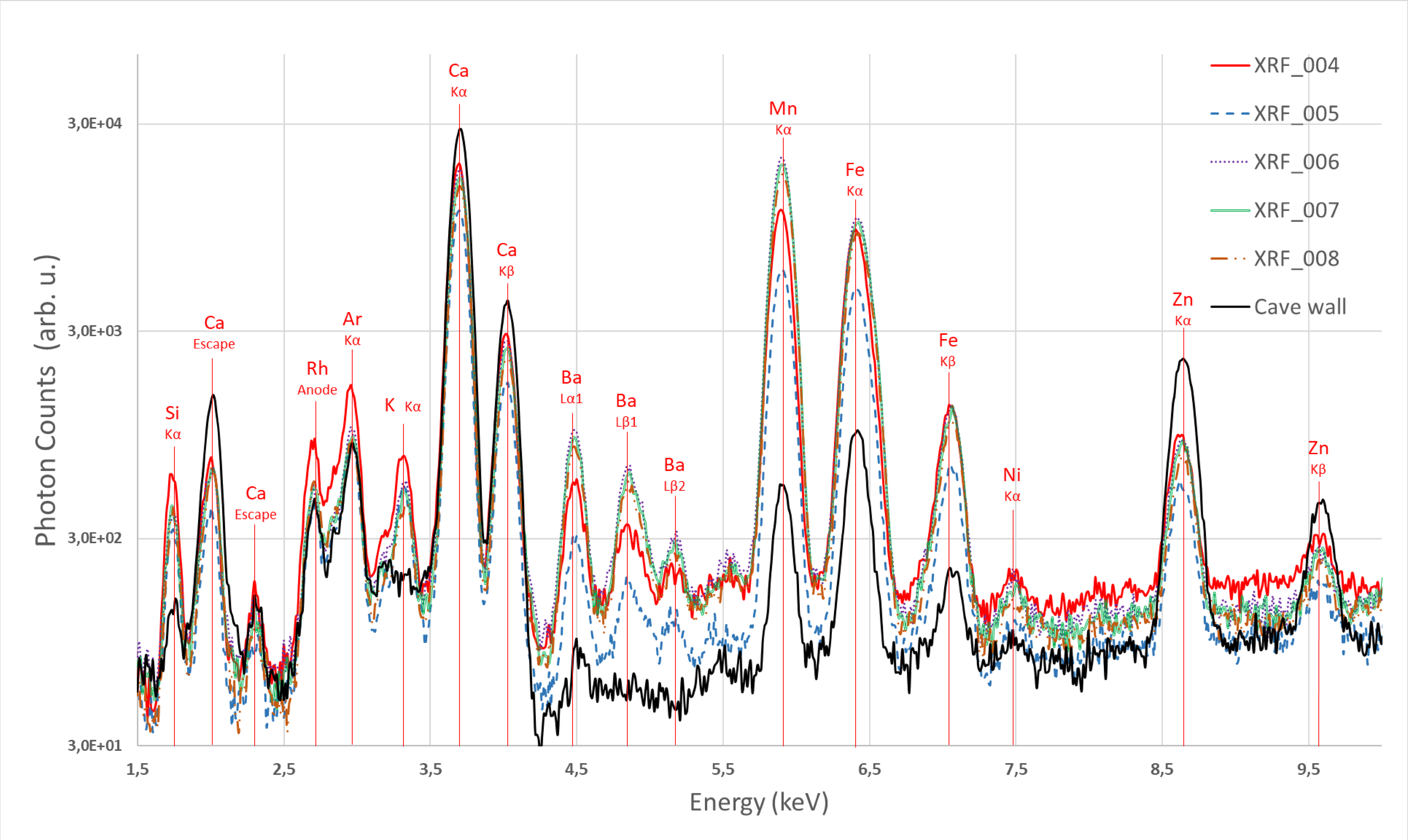

XRF Spectra

Manganese oxides
group II according to Trosseau et al. 2021

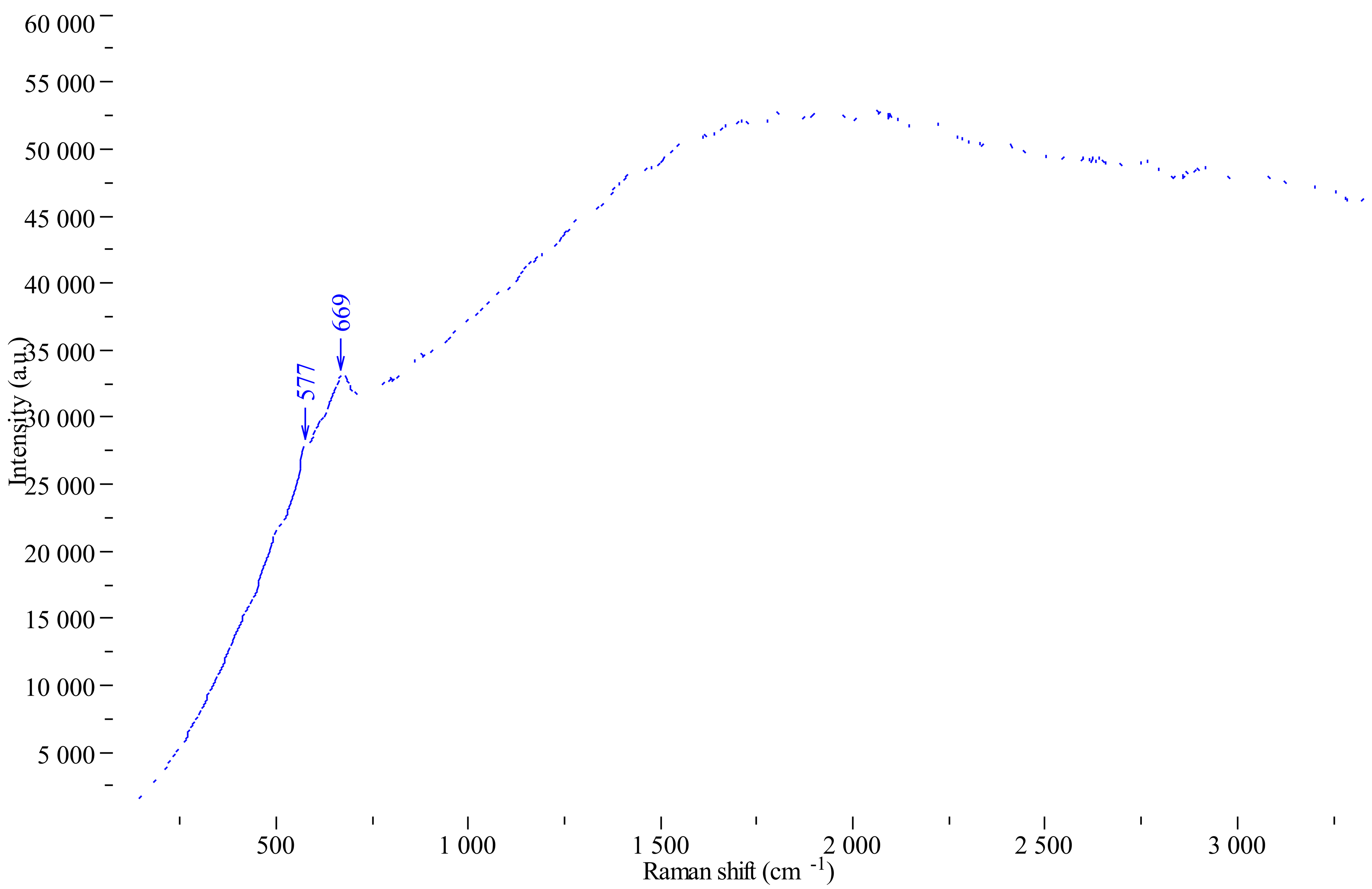

Raman Spectra

Manganese oxides
60-80% $Ba_2 Mn_5O_{10}. xH_2O$ (romanechite)
20-40% MnO_2 (pyrolusite)

Reindeer no. 12

Superimpositions
Painted and graved. Bichromic (black and red). Finely engraved details (head. antler) and scratched zones. Outlines drawn in red and black and deeply graved. Overall paint in red on a previously red area.
Represented crouched face to the reindeer of the figure 11. Directed to the left.
Lightly faded.

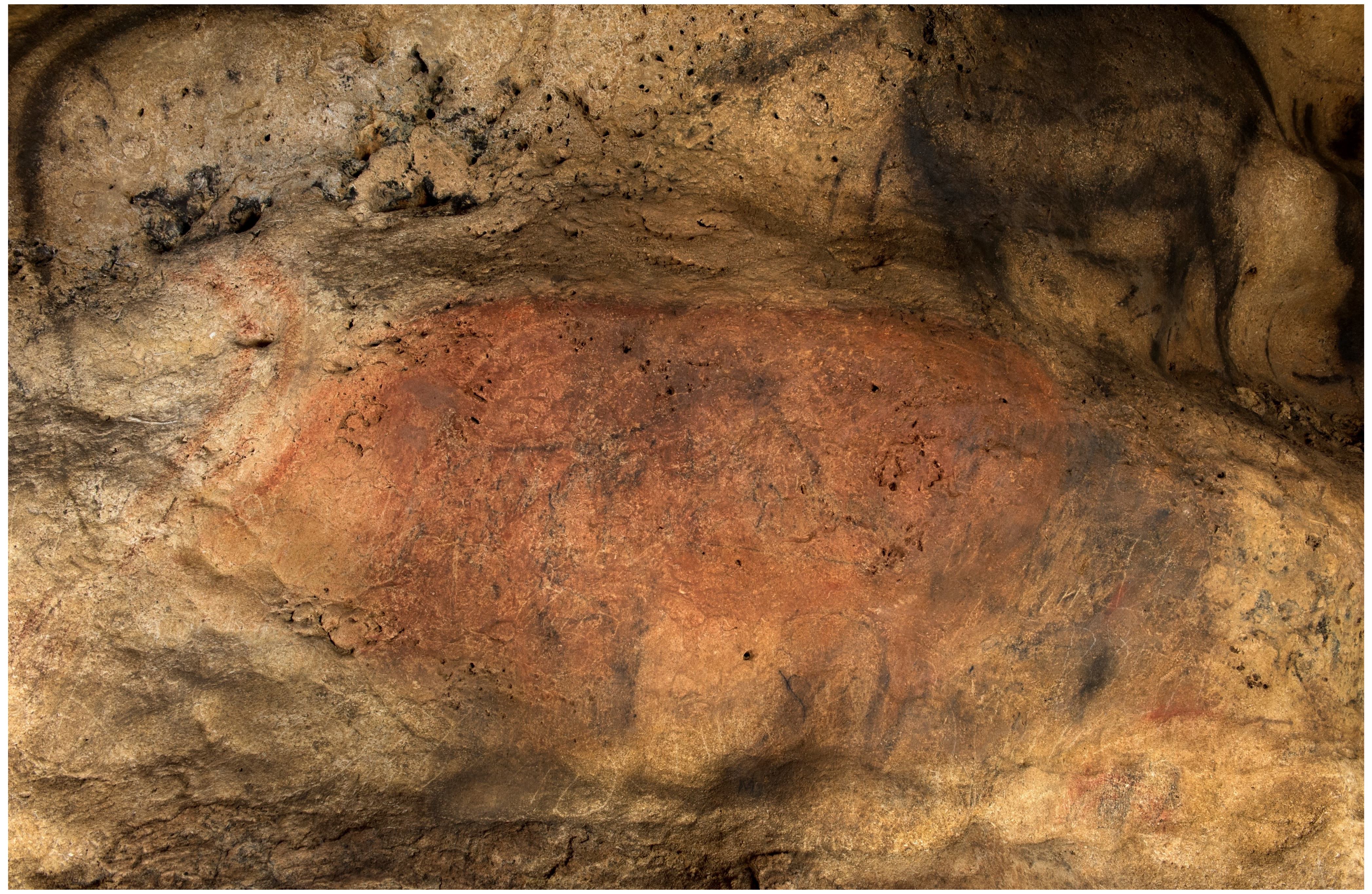

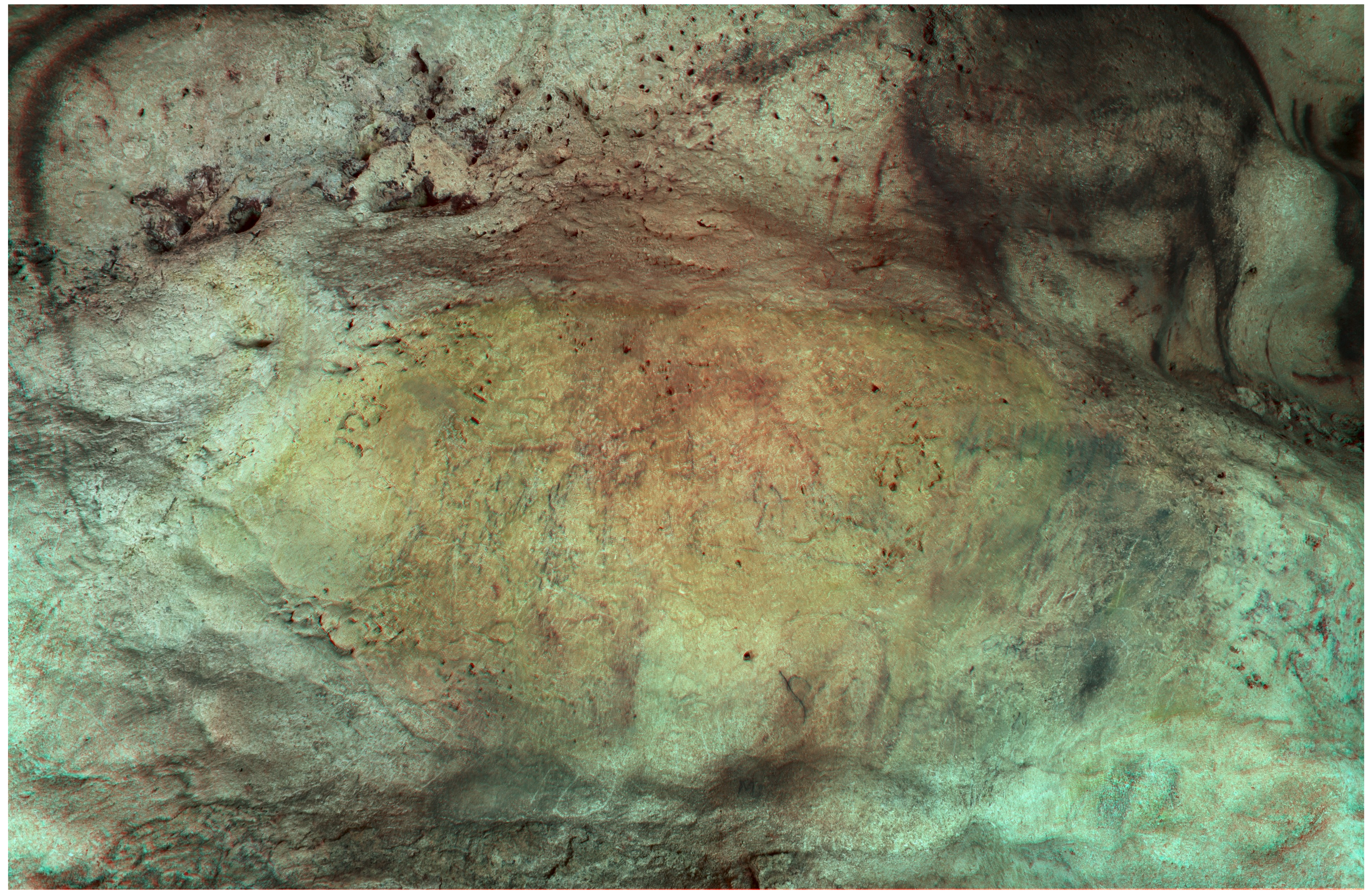

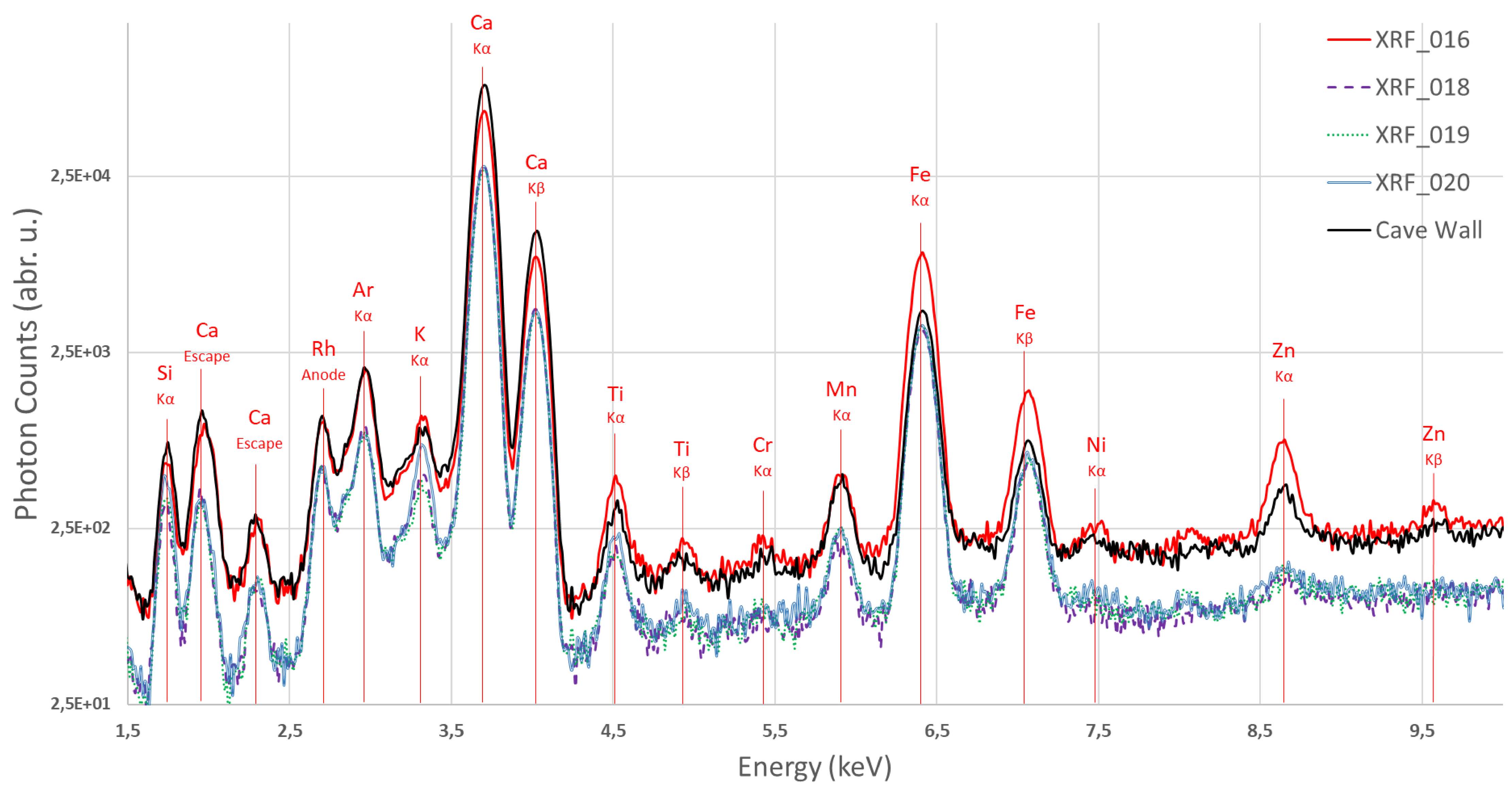

XRF spectra

Manganese oxides

No Raman spectrum

Horse no. 12

Superimposed on the Reindeer 12.
Painted in black with a plain line and graved.
Only the head is visible. Directed to the right. facing an engraved horse.

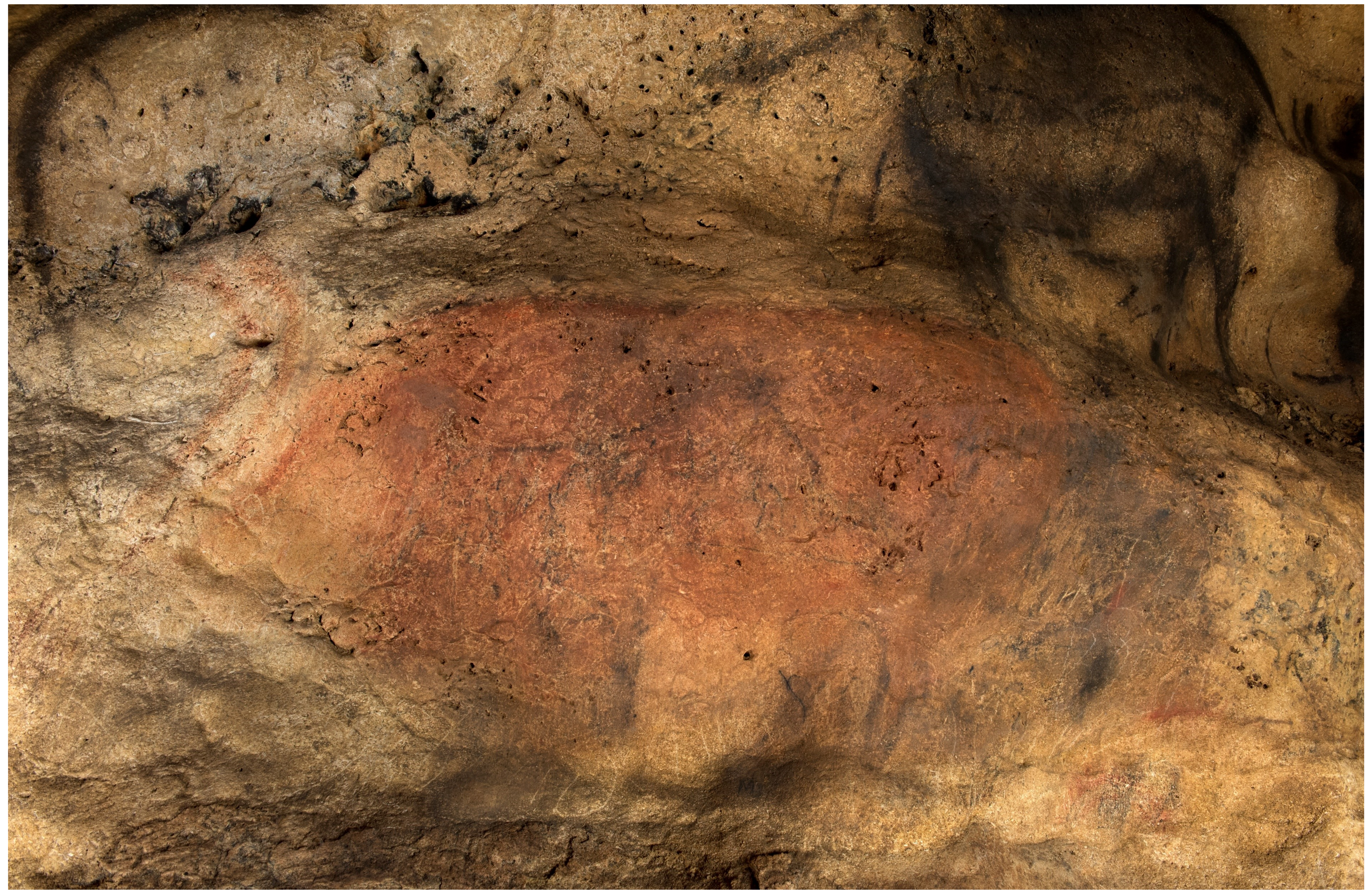

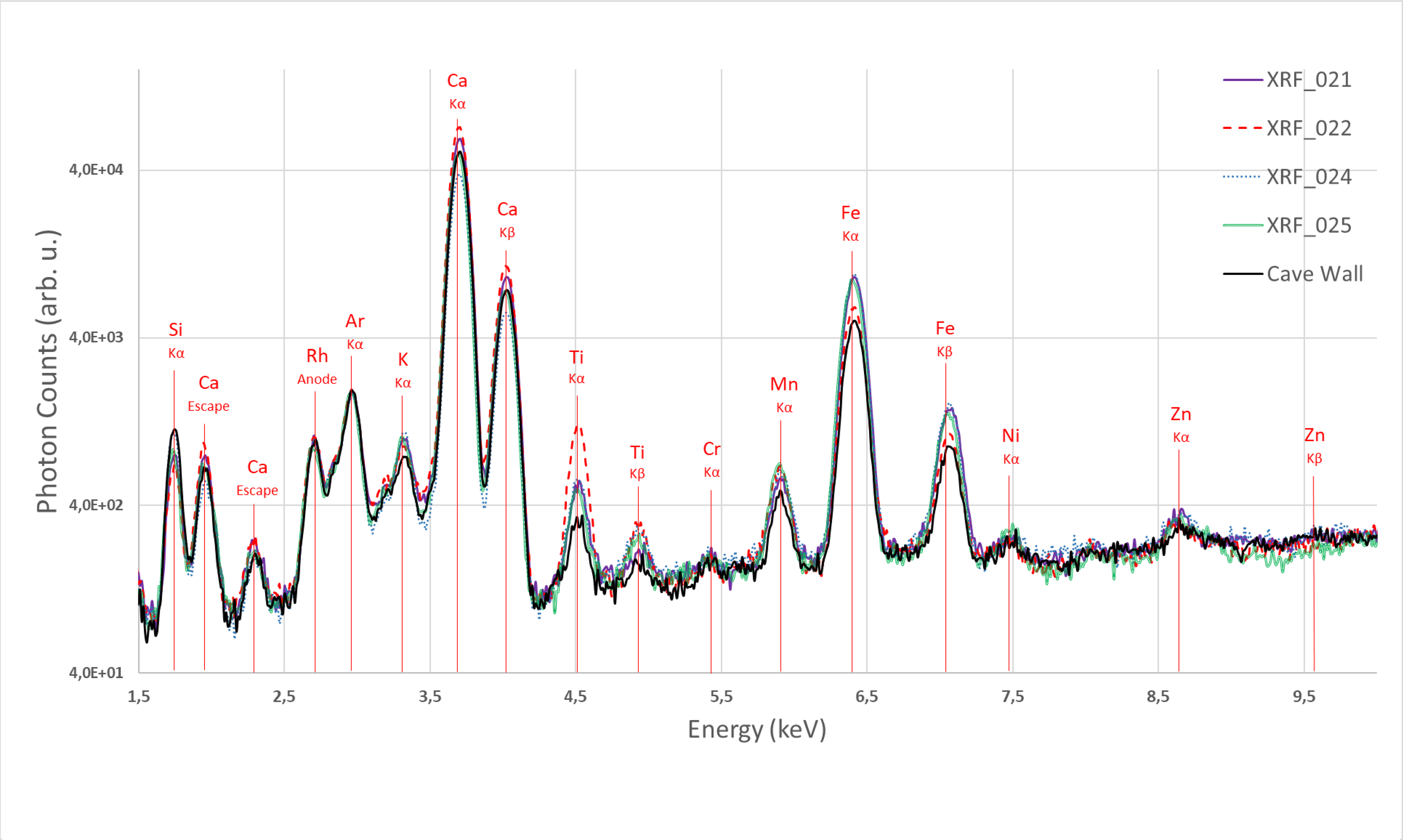

XRF spectra

Absence of Mn

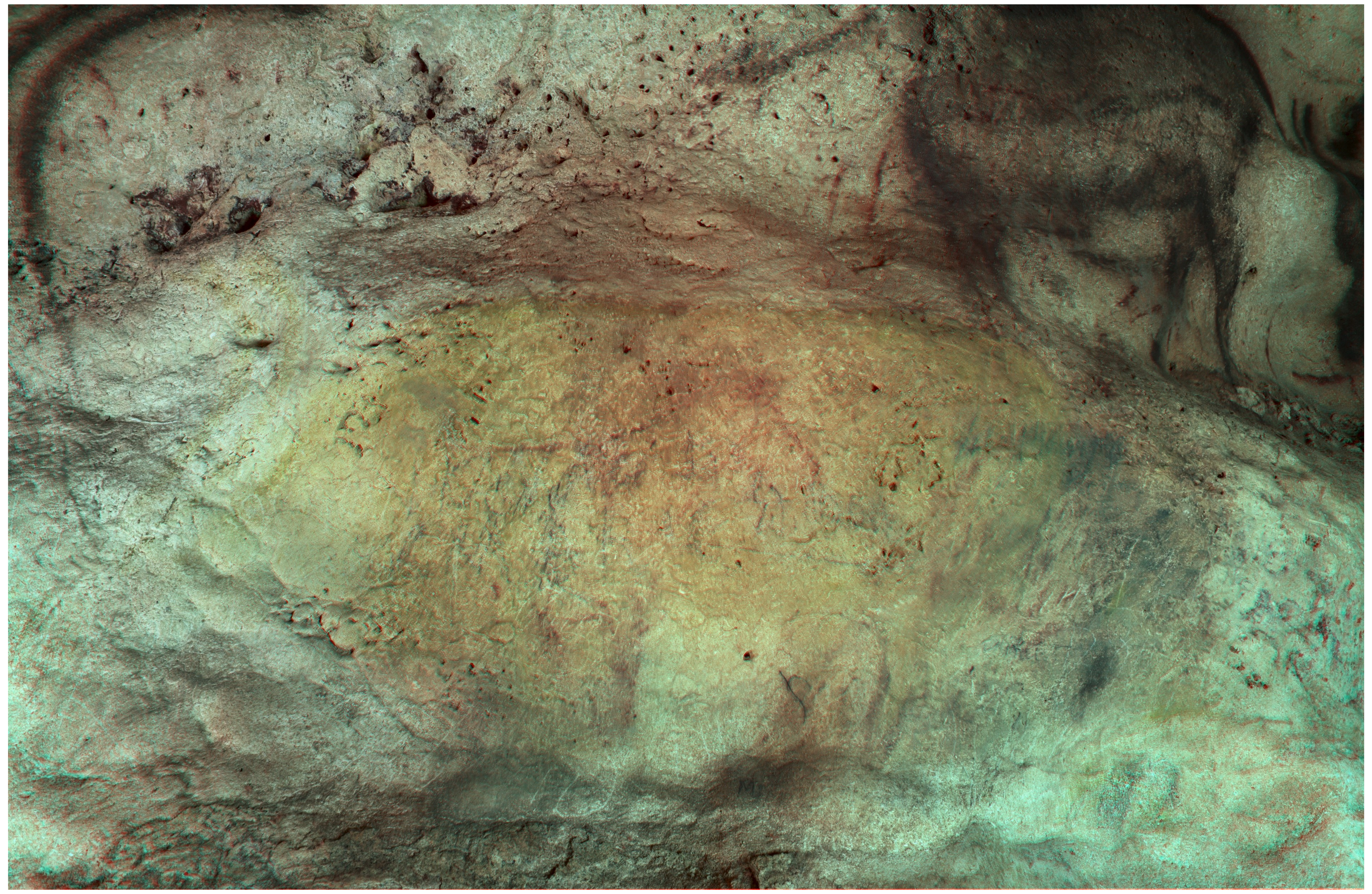

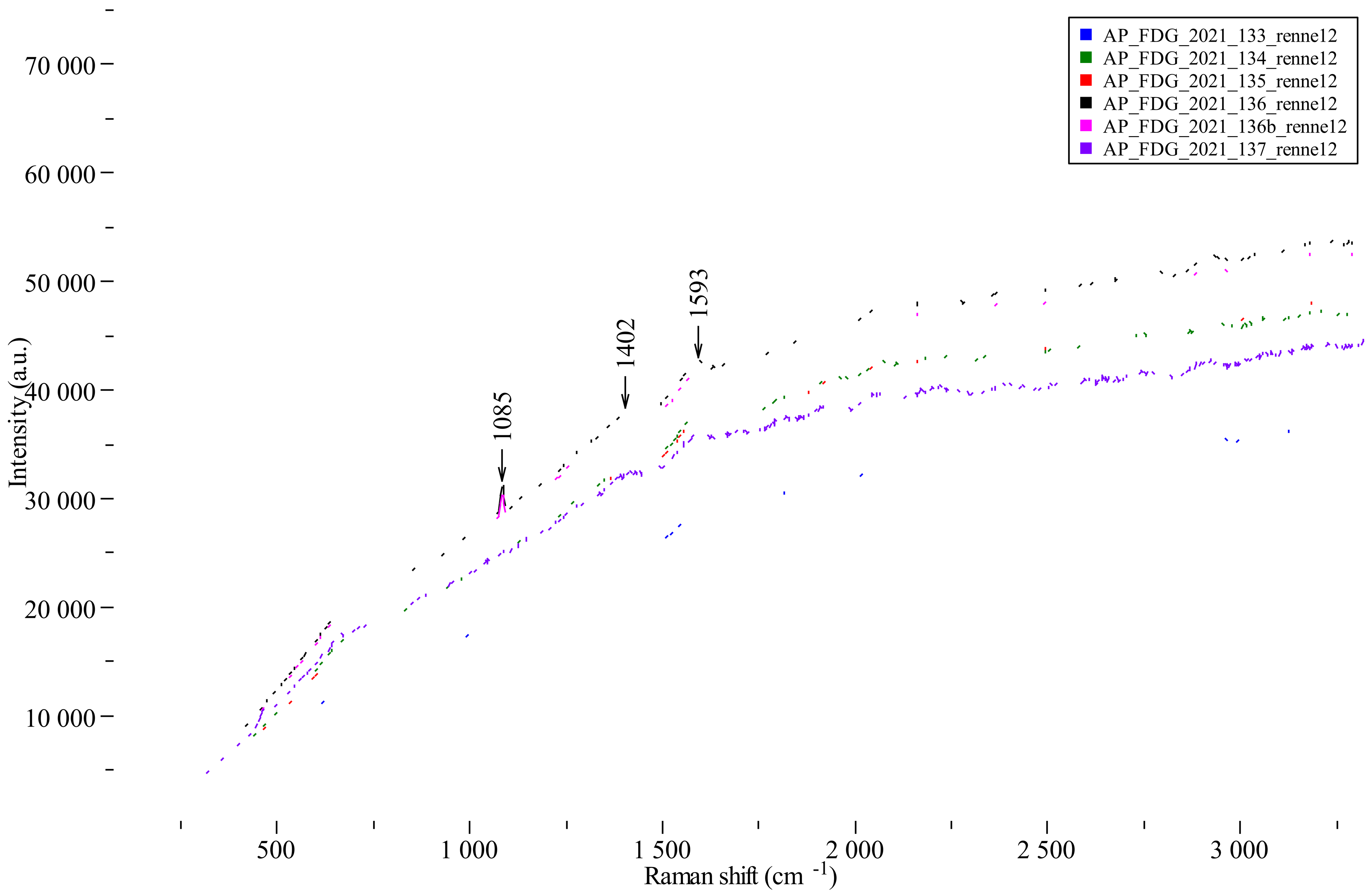

Raman spectra

Charcoal

Deer no. 13

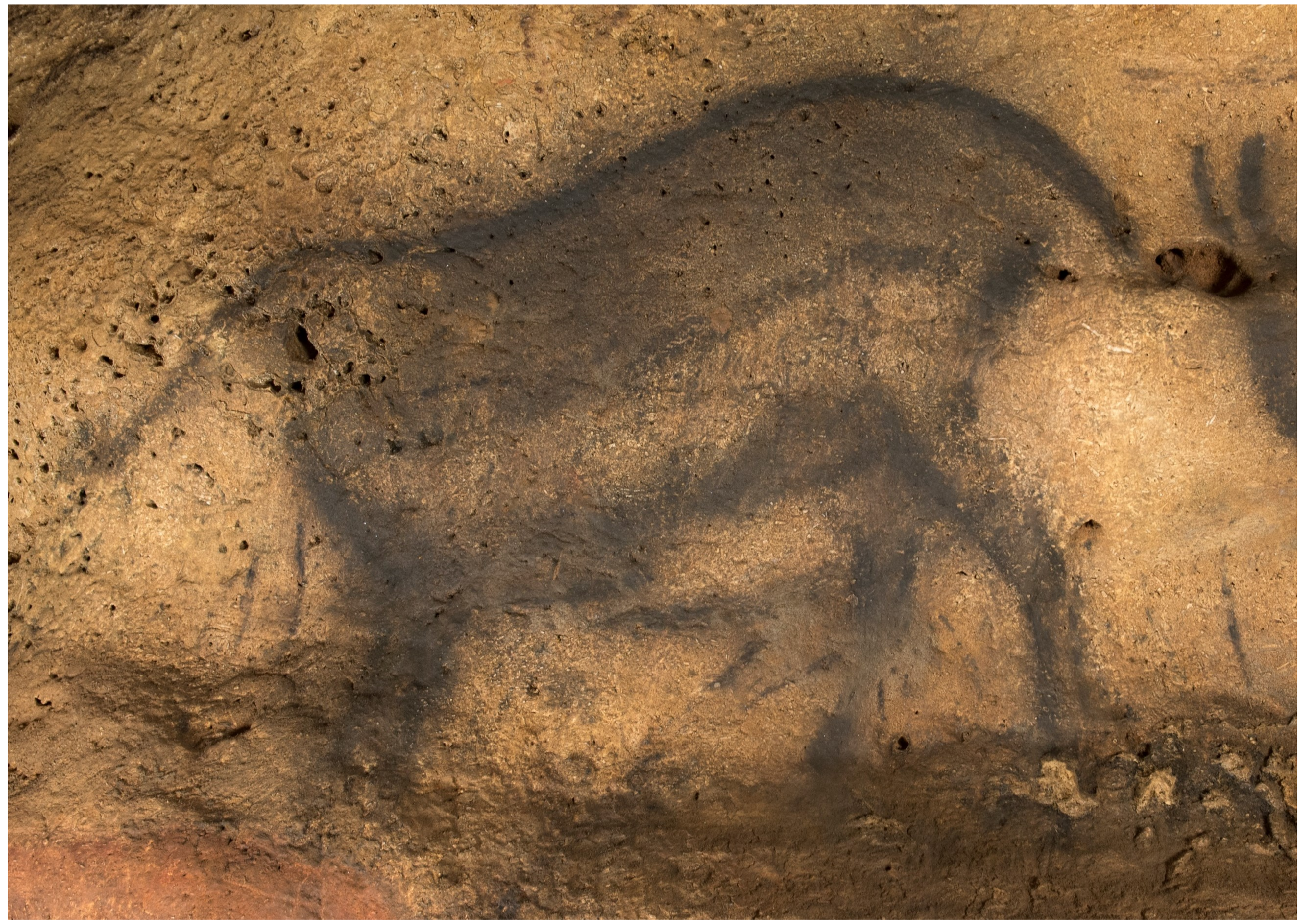

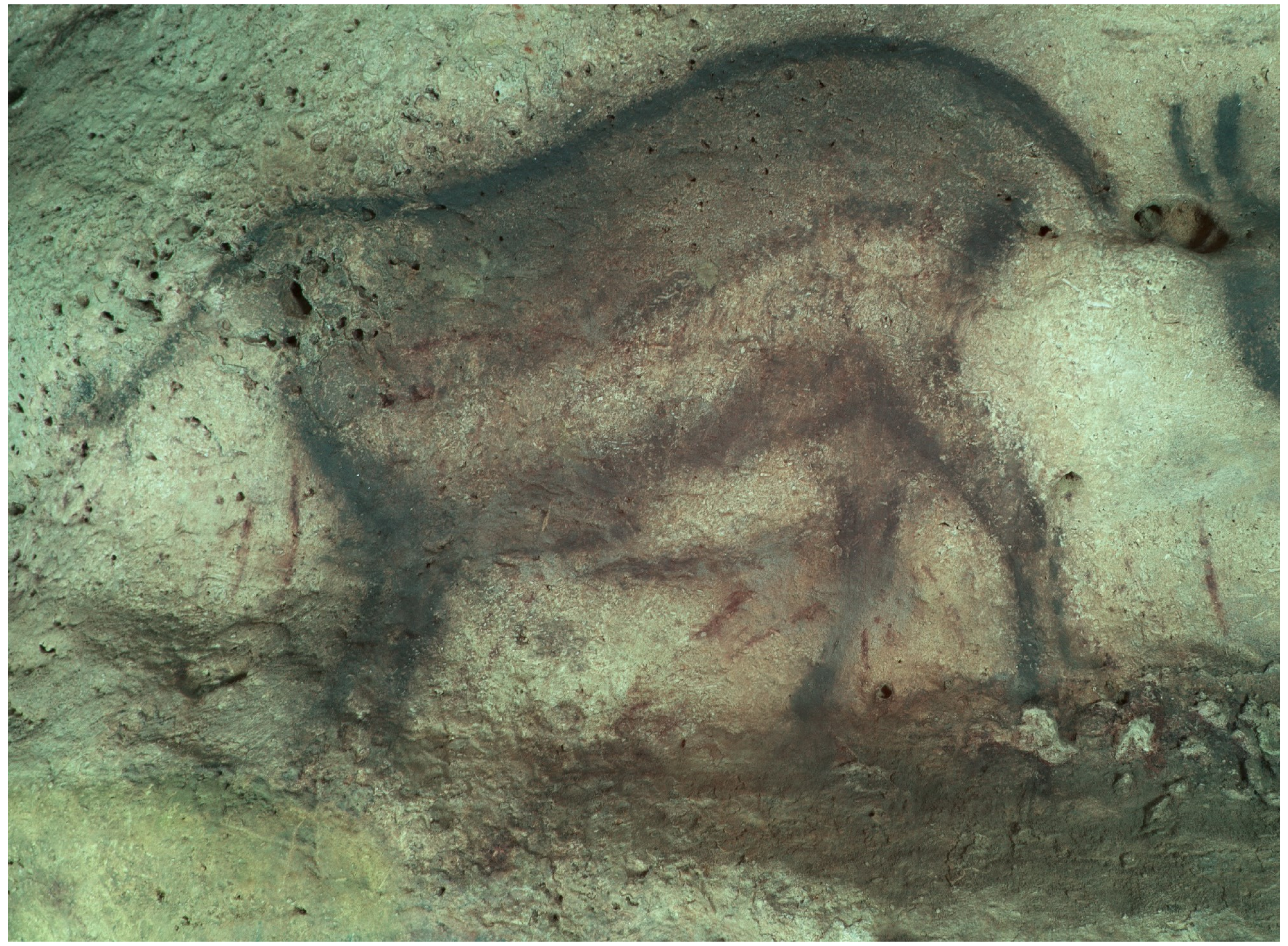

Superimposed to an engraved horse.
Painted in black and finely graved. Outlines drawn in thick black and partly graved and inner painted with gradient. The head could be graved.
Directed to the left. First animal of a black frieze.
Well preserved except for the head.

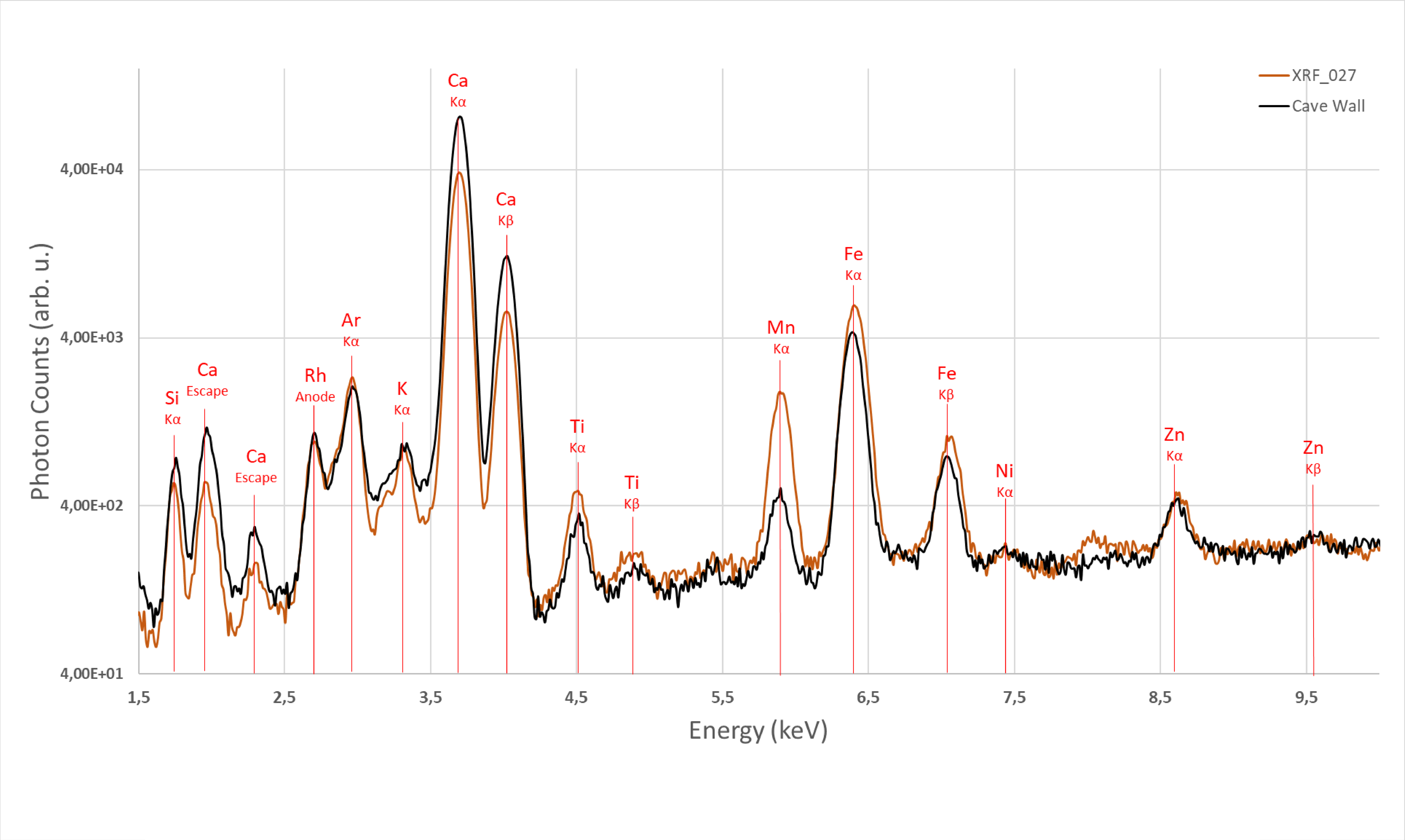

XRF spectra

Manganese oxide

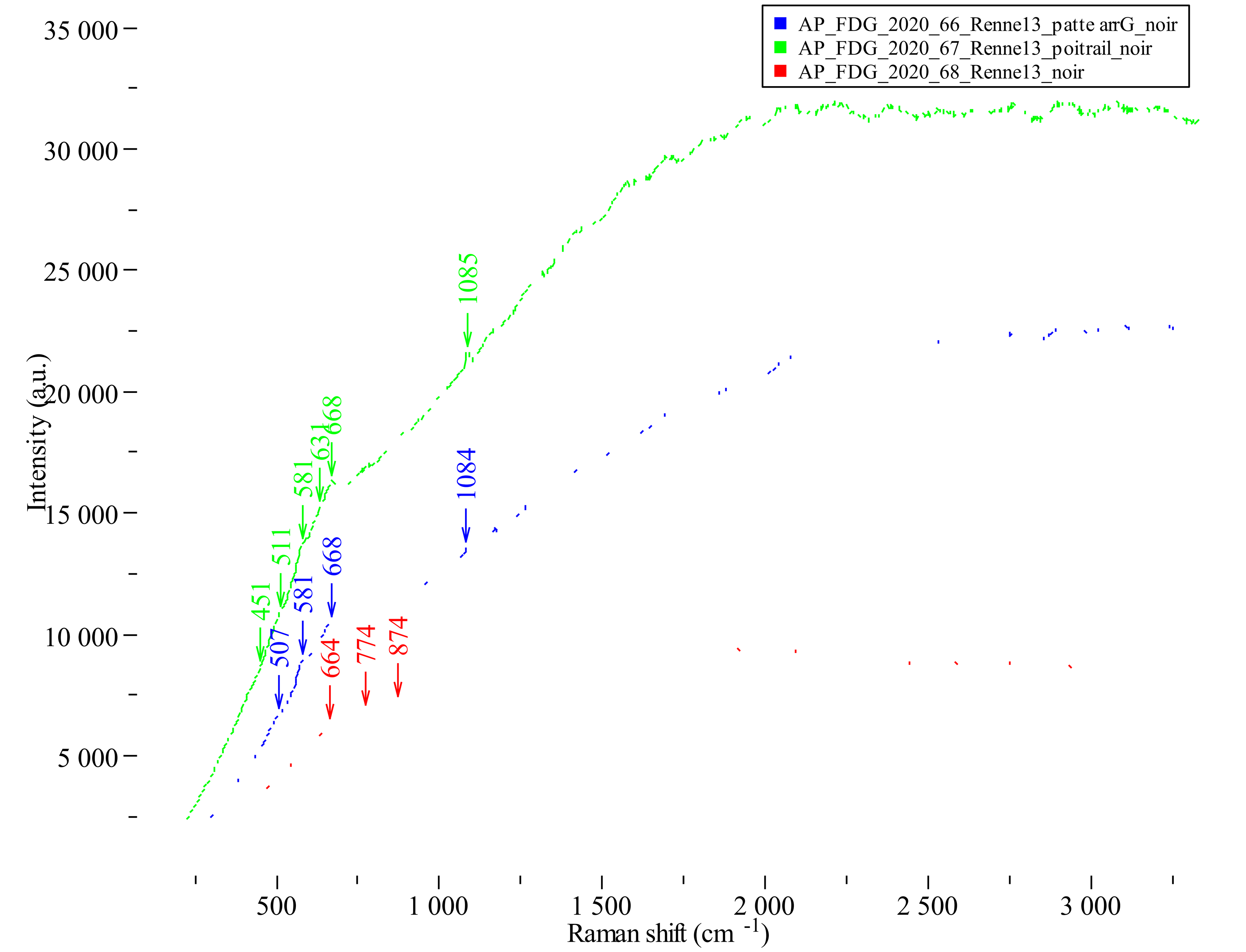

Raman spectra

Manganese oxides
charcoal

Dots near Deer no. 13

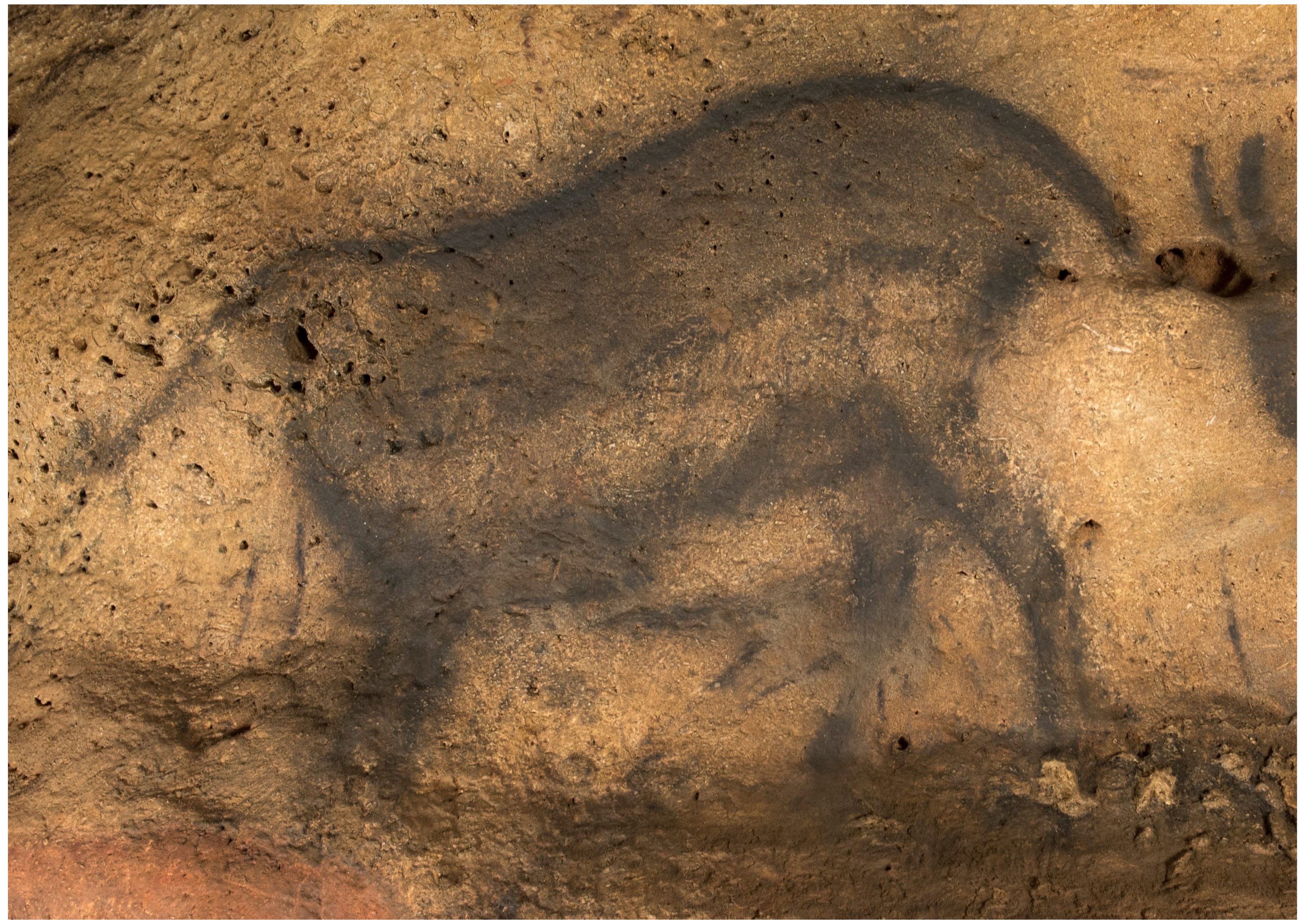

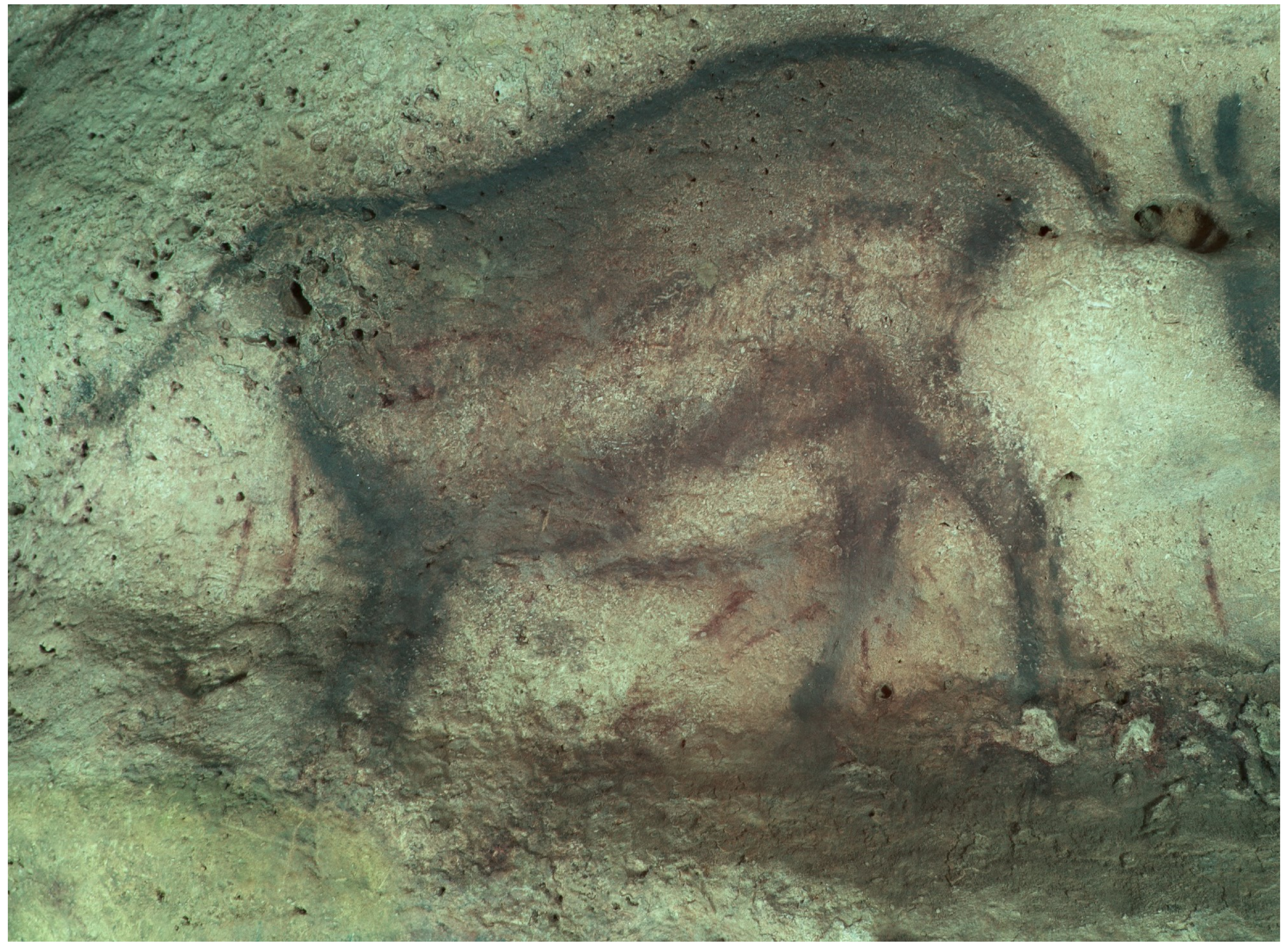

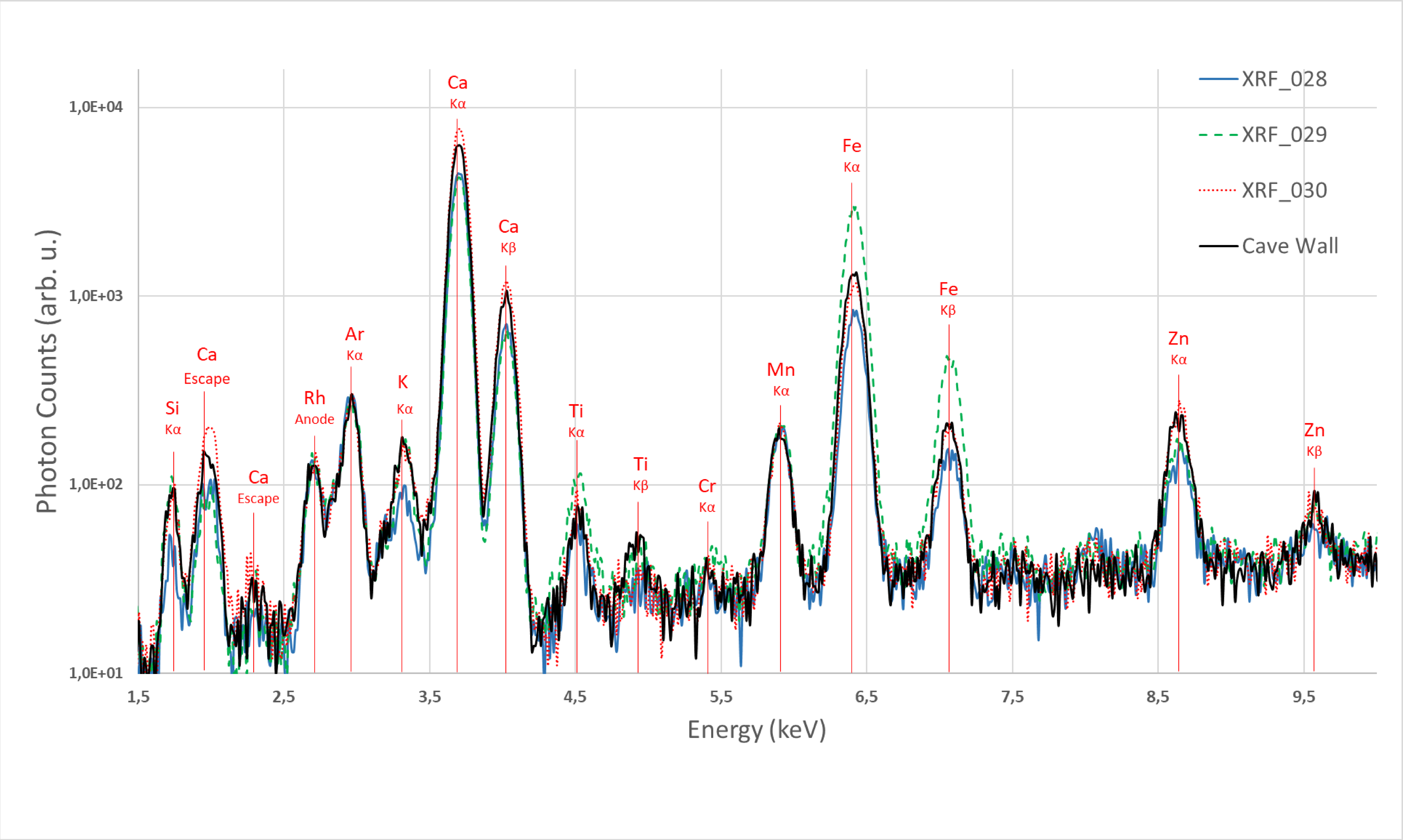

XRF spectra

Absence of Mn

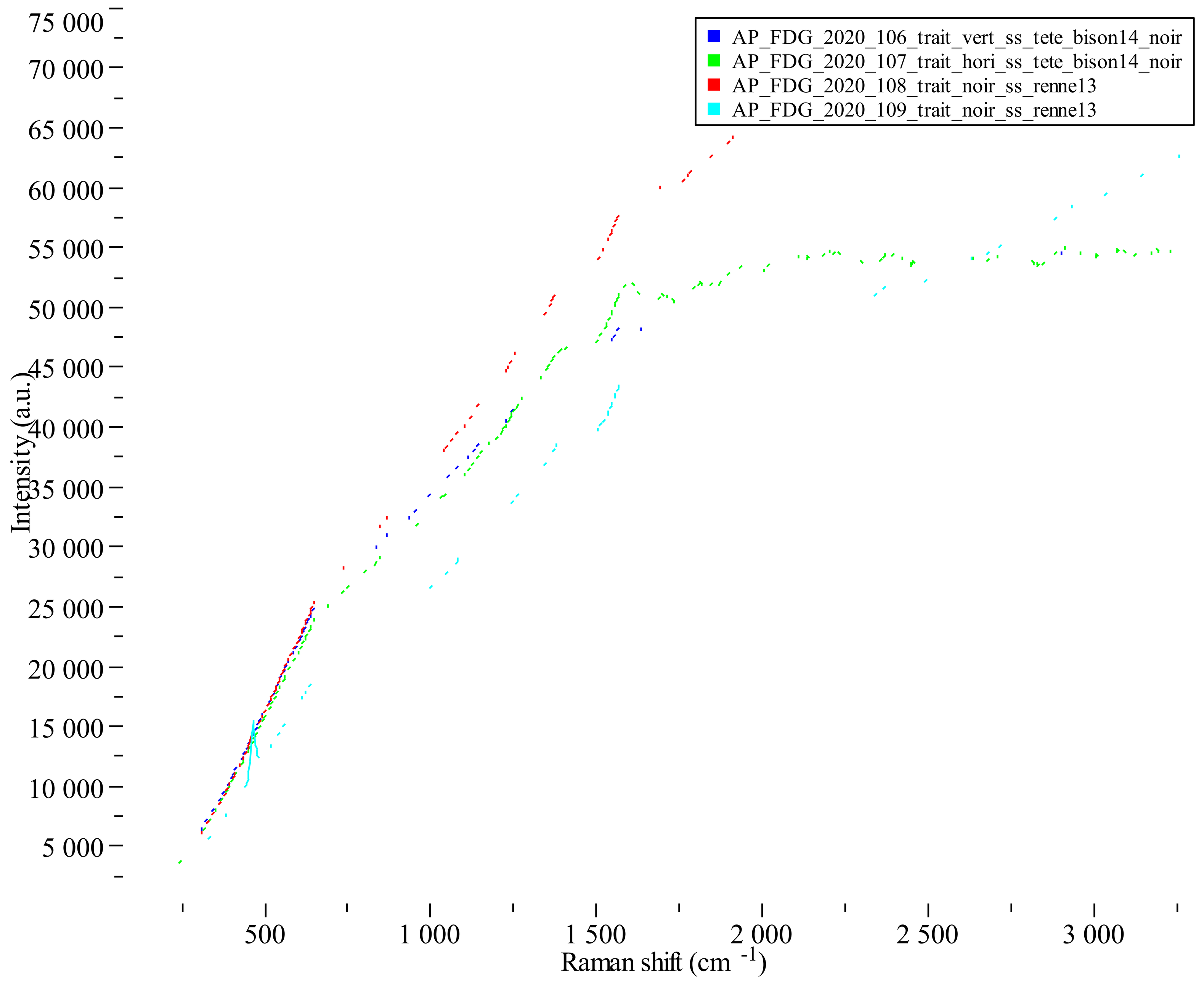

Raman spectra

charcoal

Bison 14 and black dots

Superimpositions
Drawn in black and partly graved.
Following the reindeer of the figure 13, the back is not represented.
Well preserved.

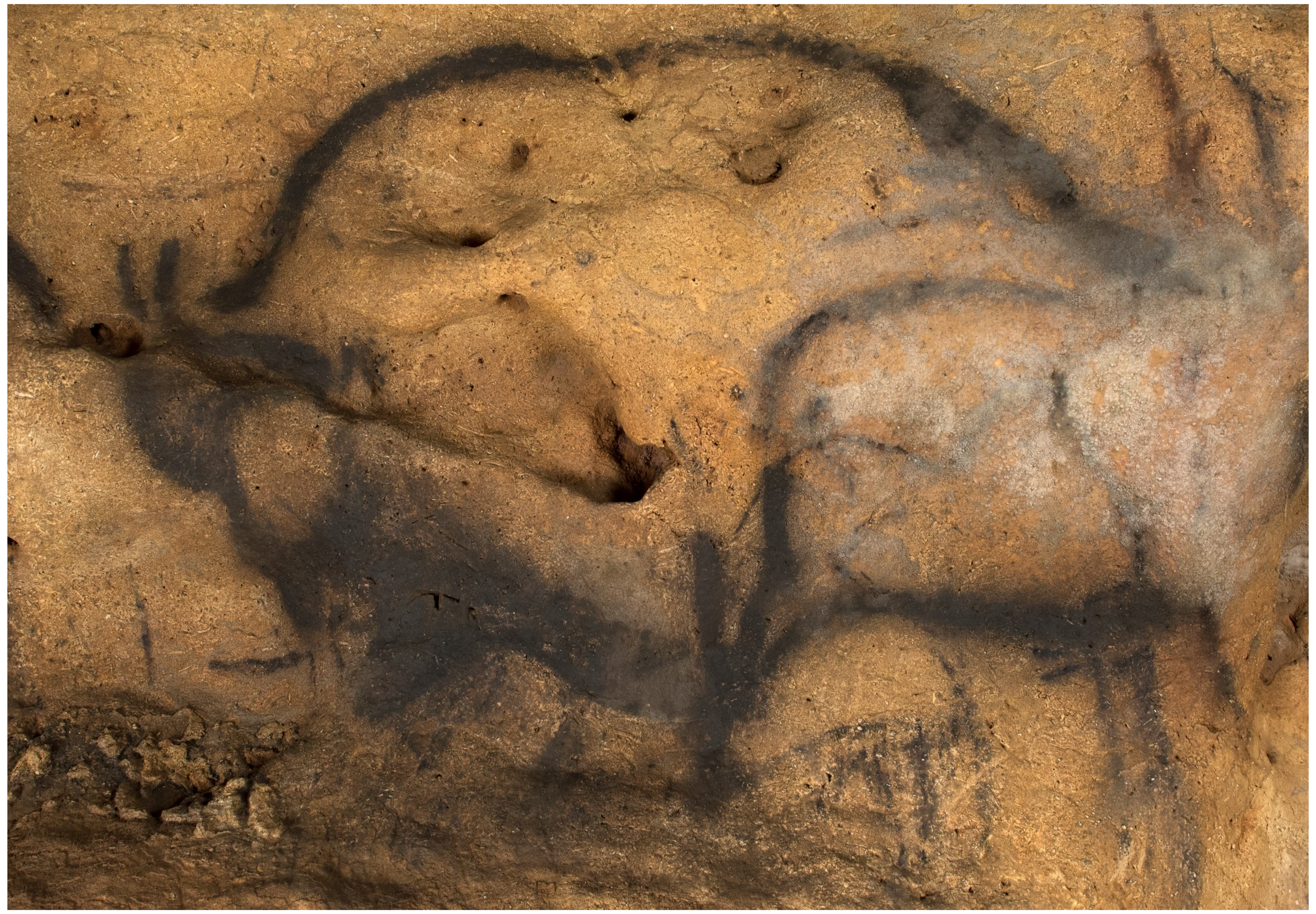

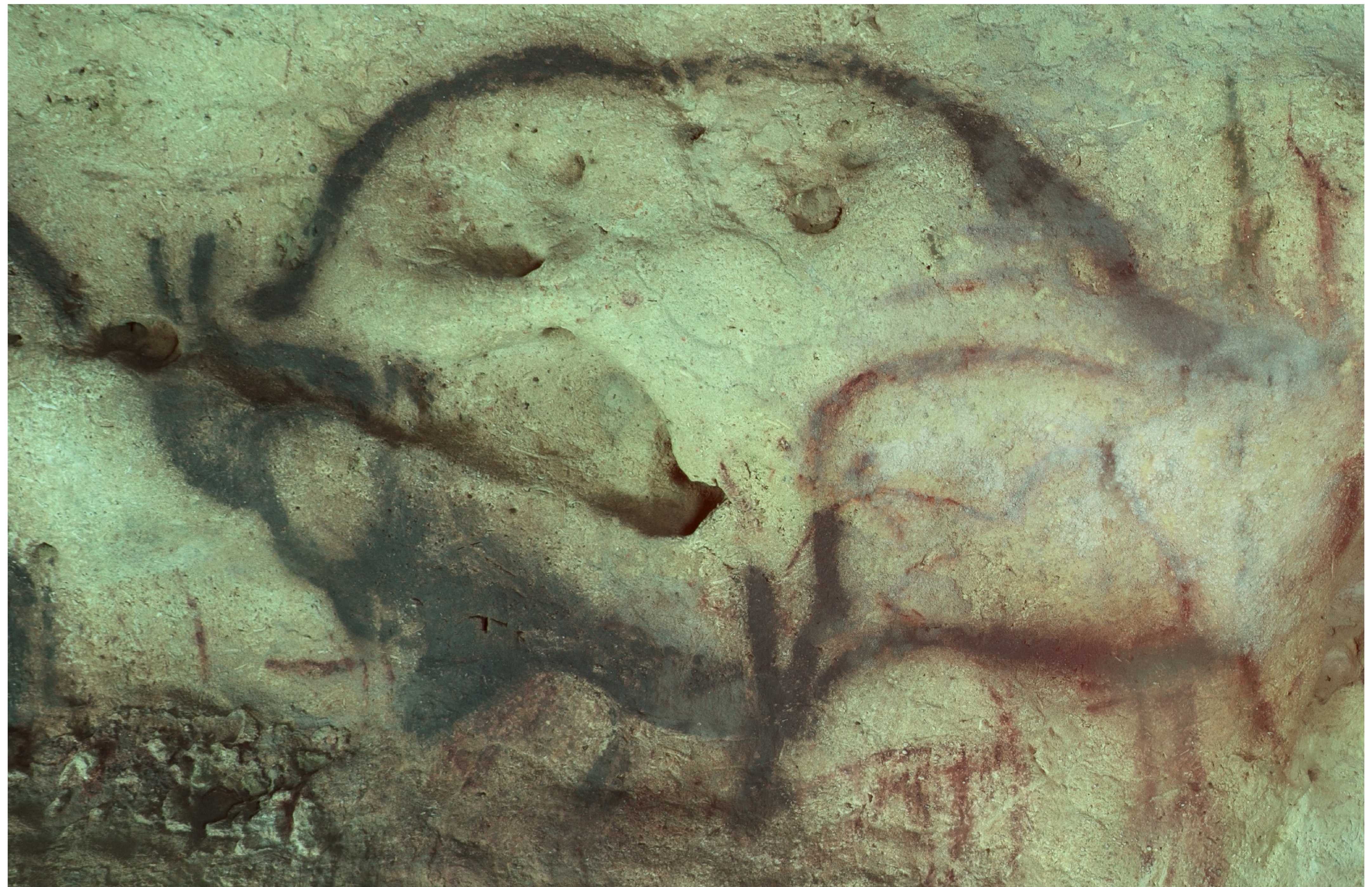

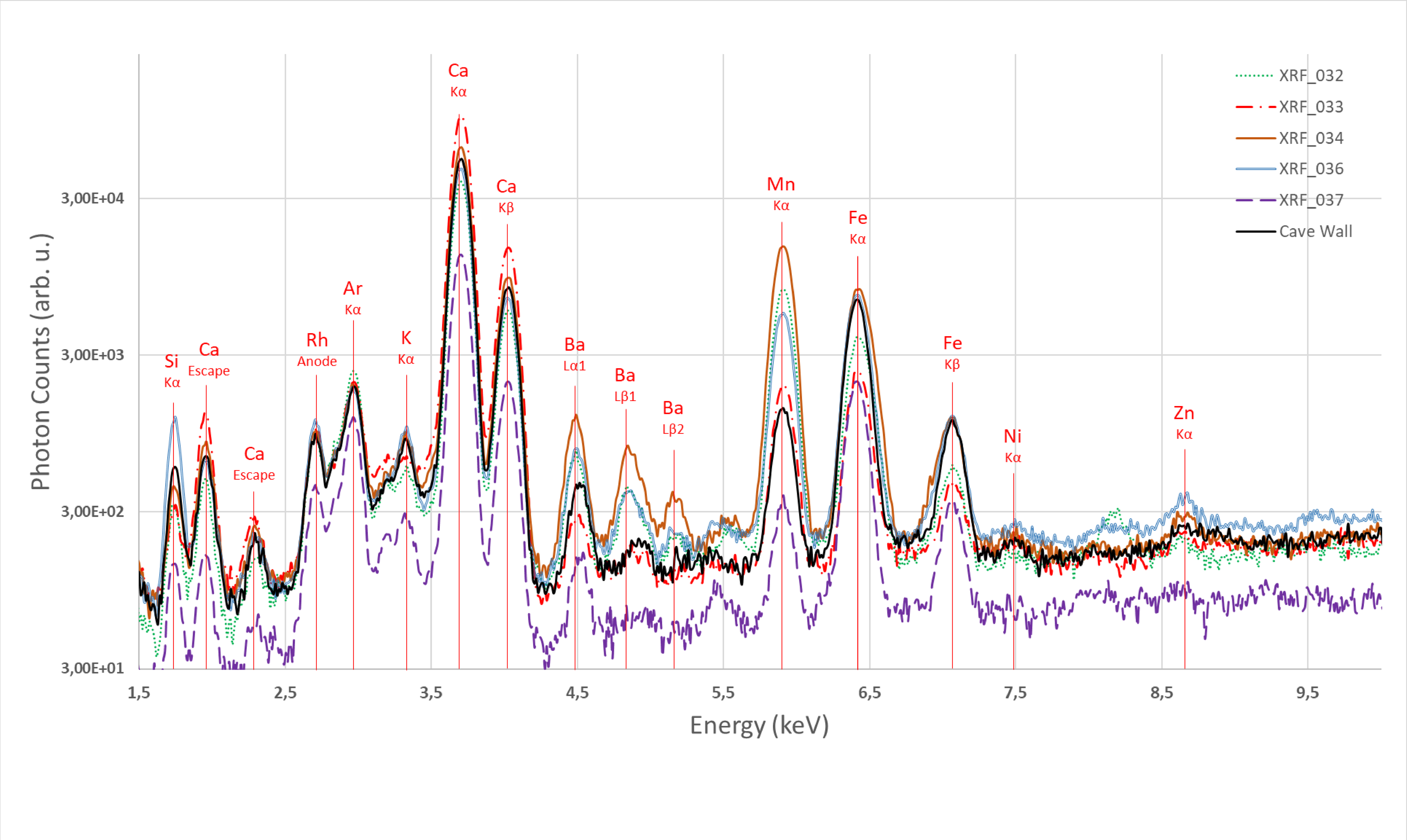

XRF spectra

Manganese oxides
group I according to Trosseau et al. 2021

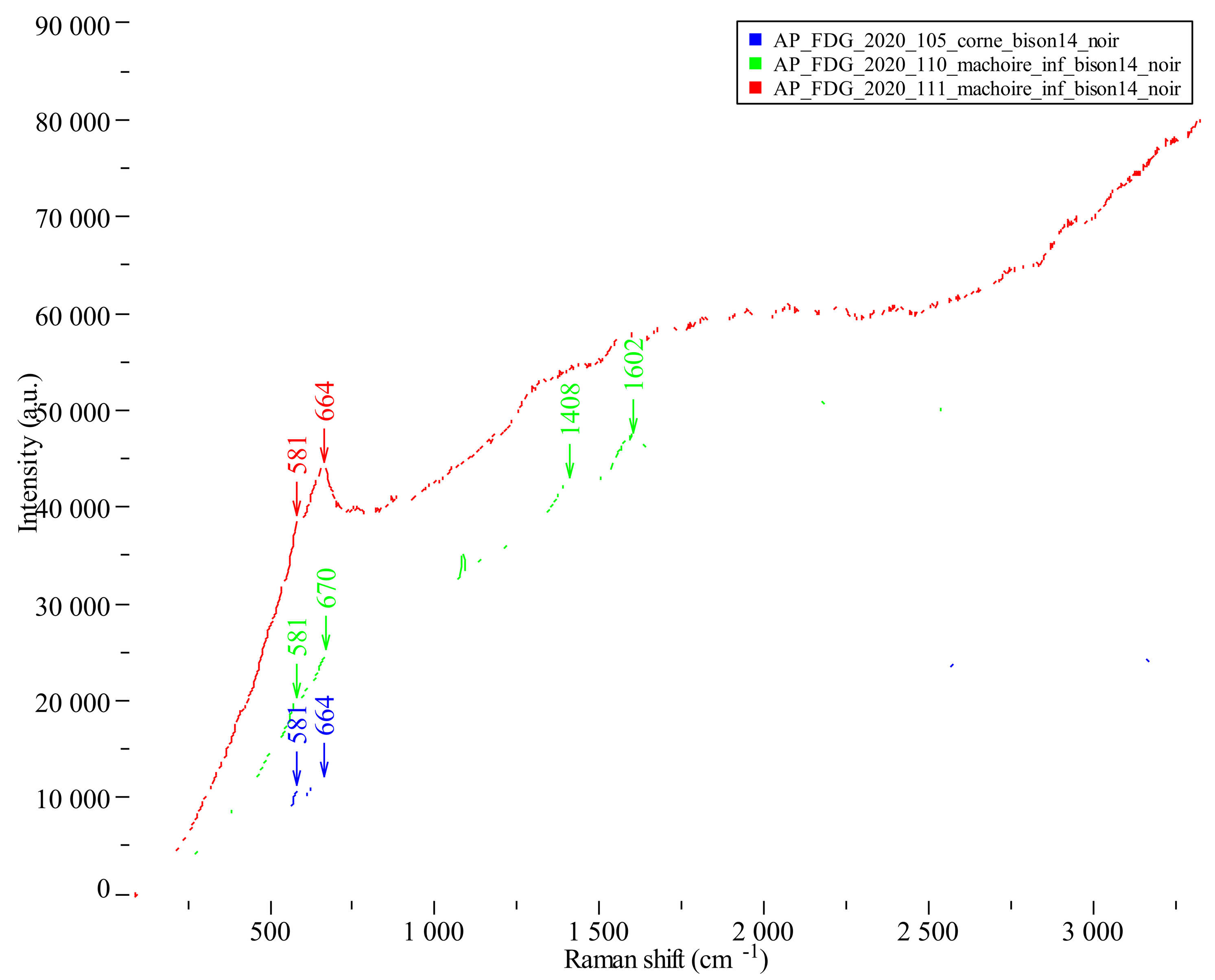

Raman spectra

Manganese oxides
80-90% $Ba_2Mn_5O_{10}$, xH_2O (romanechite)
10-20% MnO_2 (pyrolusite)

charcoal

Other Figure, possibly a Deer 14

Superimposed on the Bison 14.
Drawn in black.
Shares its back leg with the bison. Seems turned in three-quarter perspective.
Well preserved but covered with calcite veil.

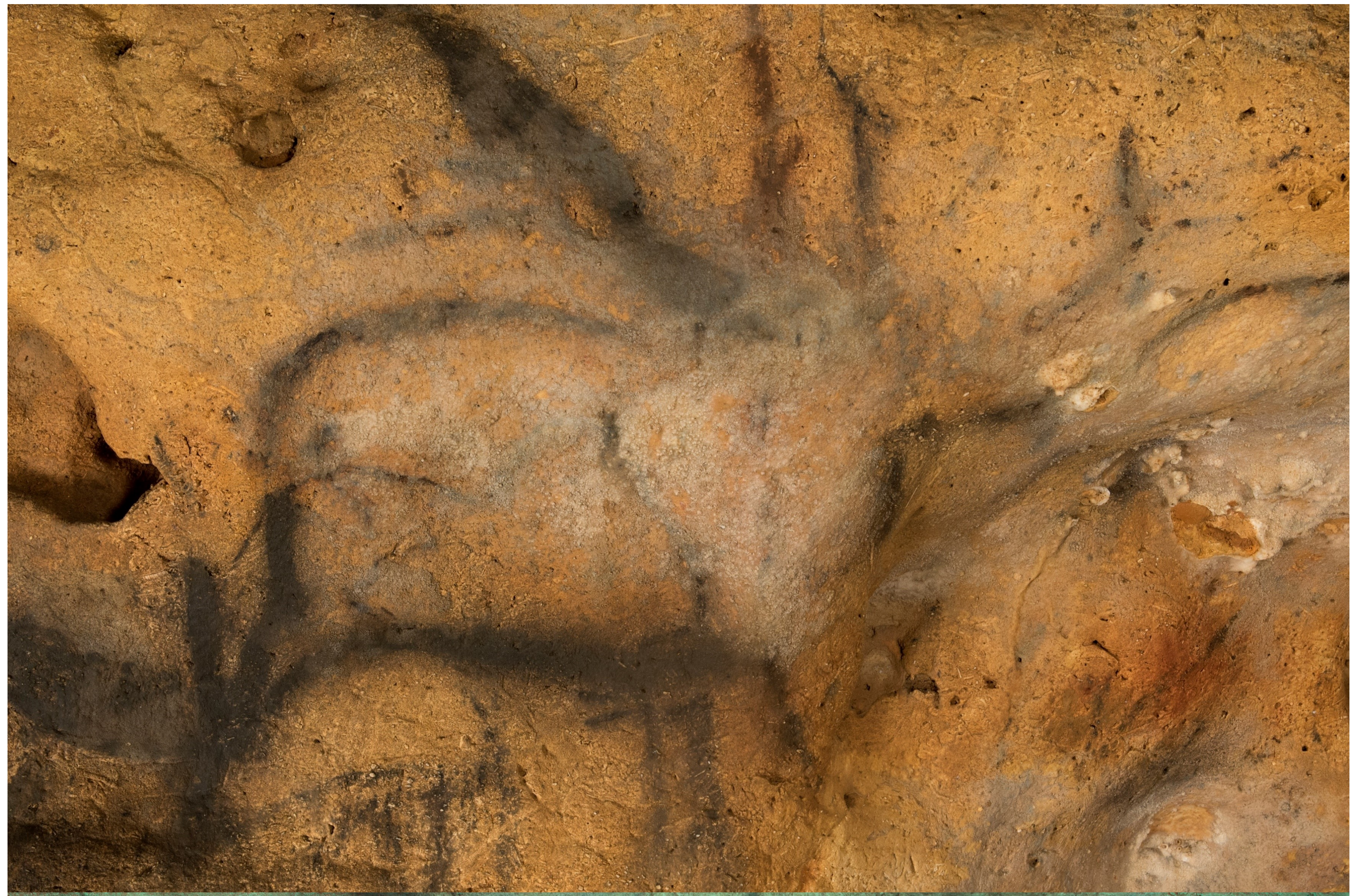

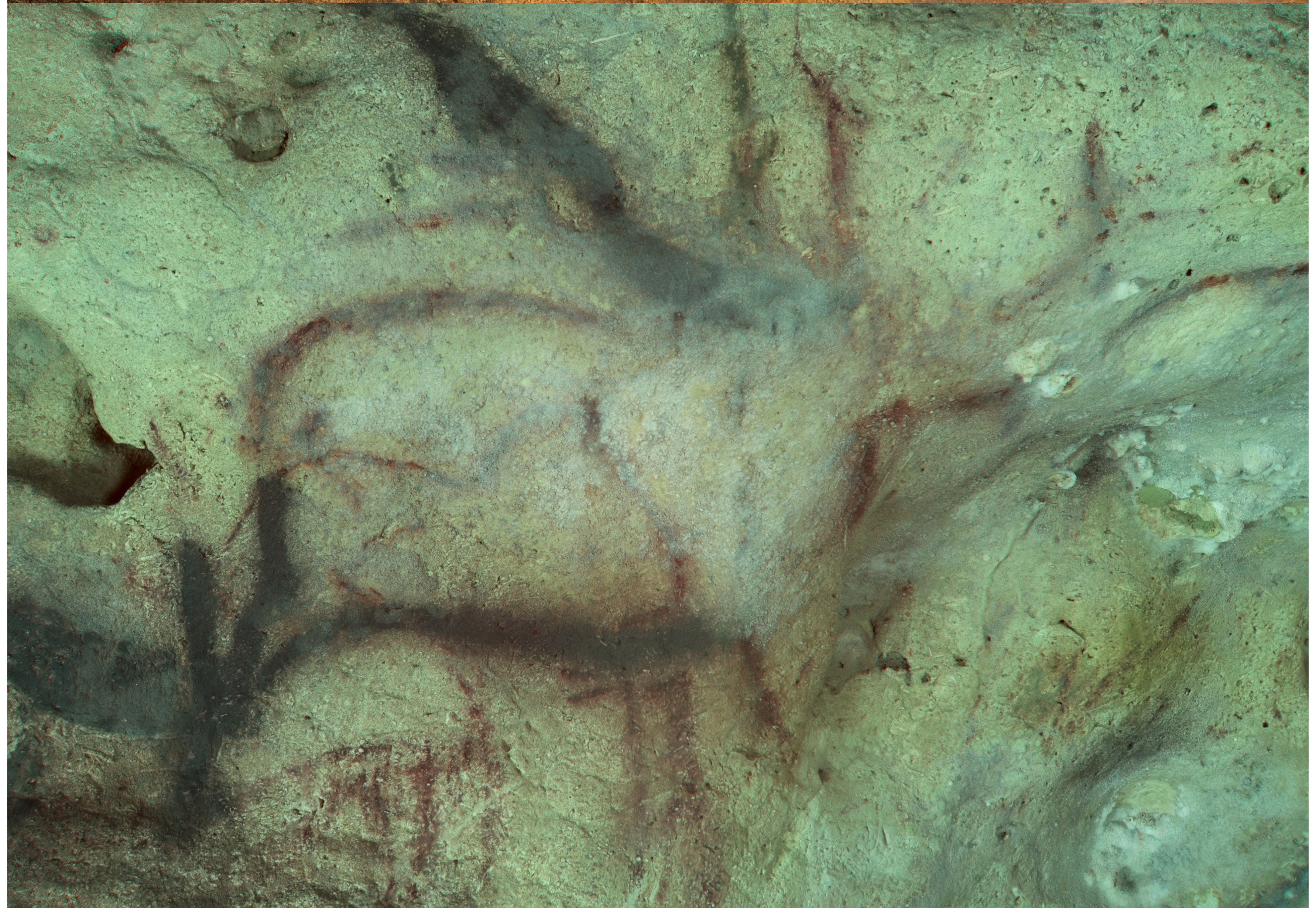

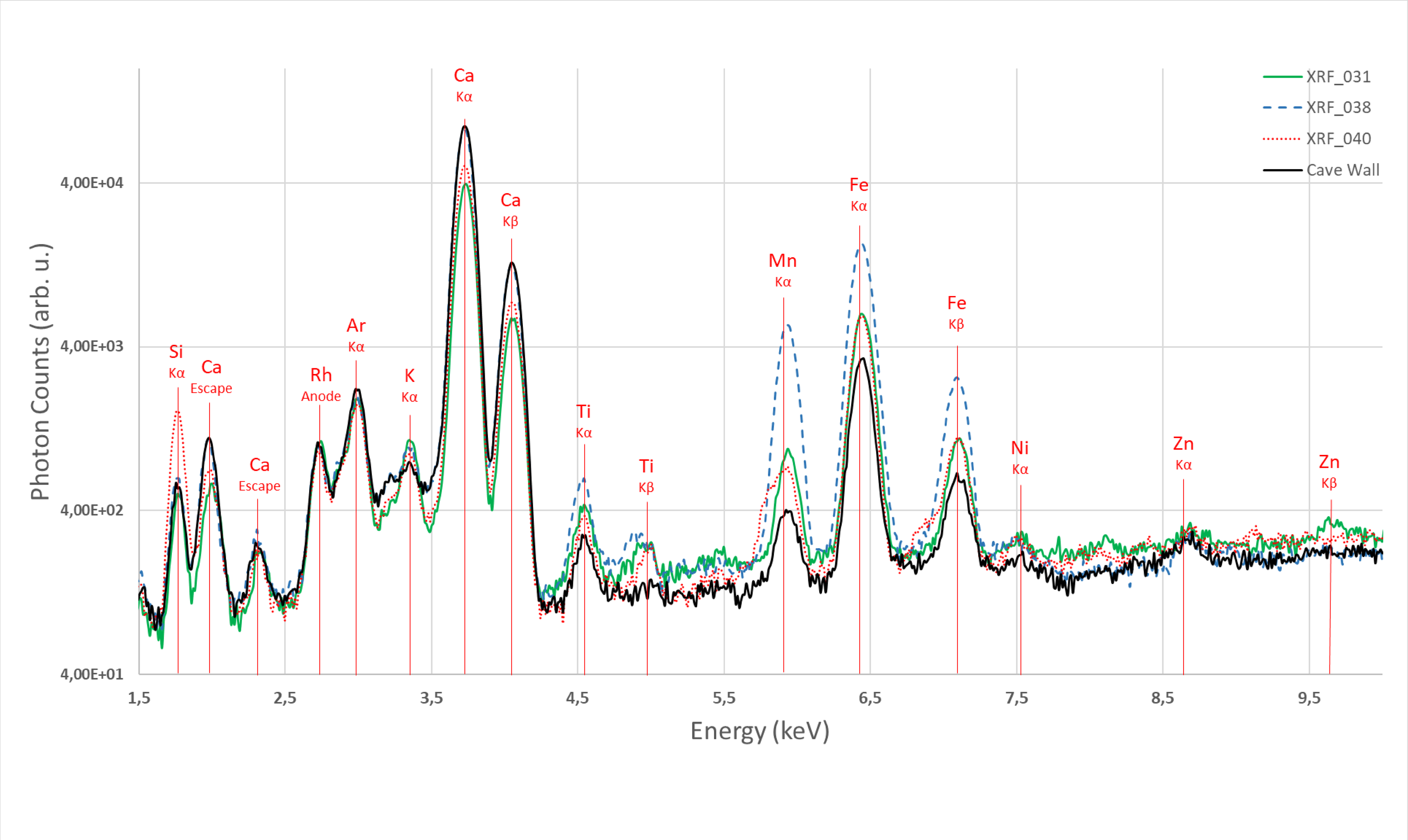

XRF spectra

Absence of Mn

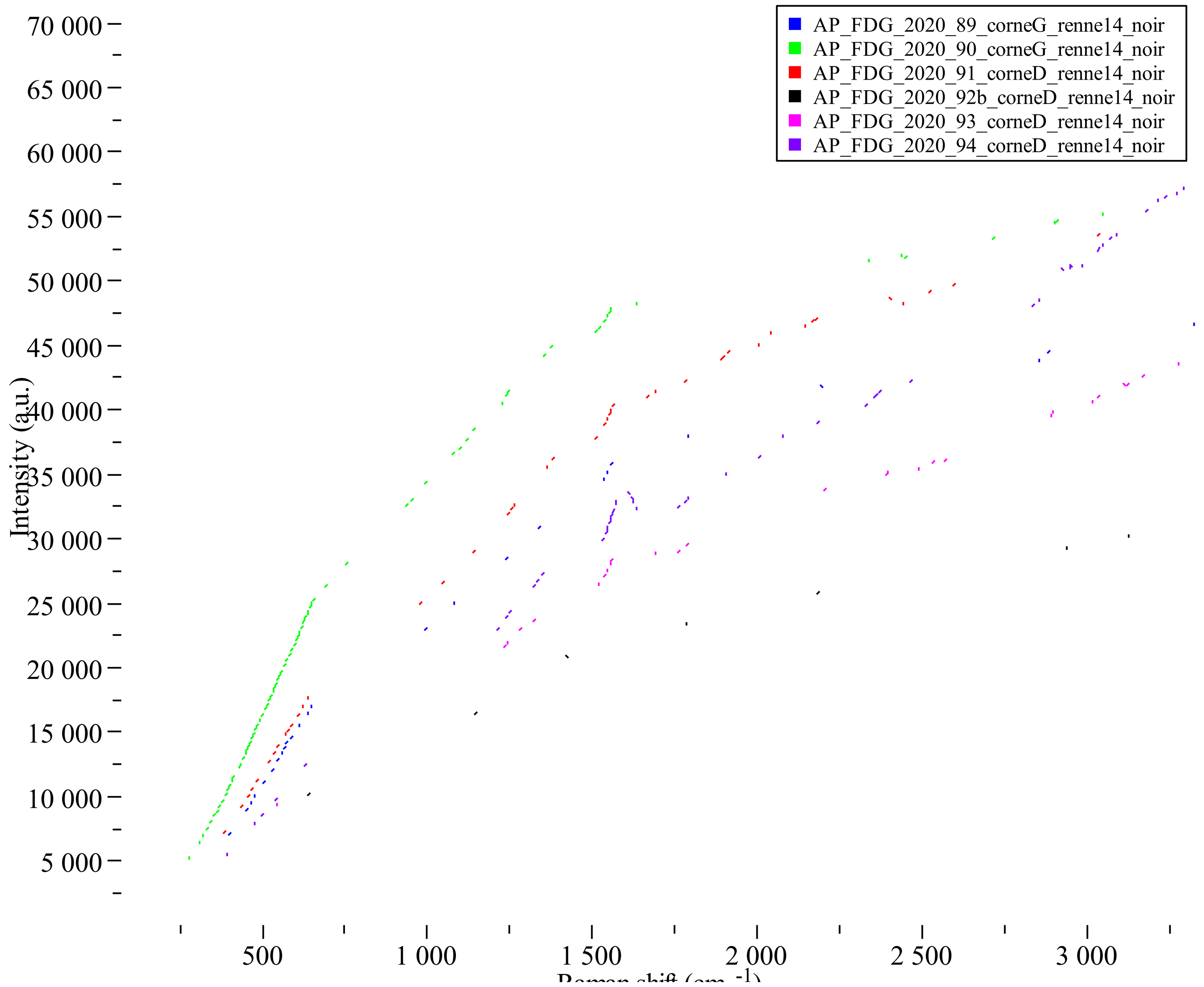

Raman spectra

charcoal

Bison 15

No superimposition
Profile drawn in black. Figurative horns in half-twisted perspective
Drawn in black and partly graved.
Directed to the right, but the head is turned in a front perspective.
Well preserved. – Calcite veil.

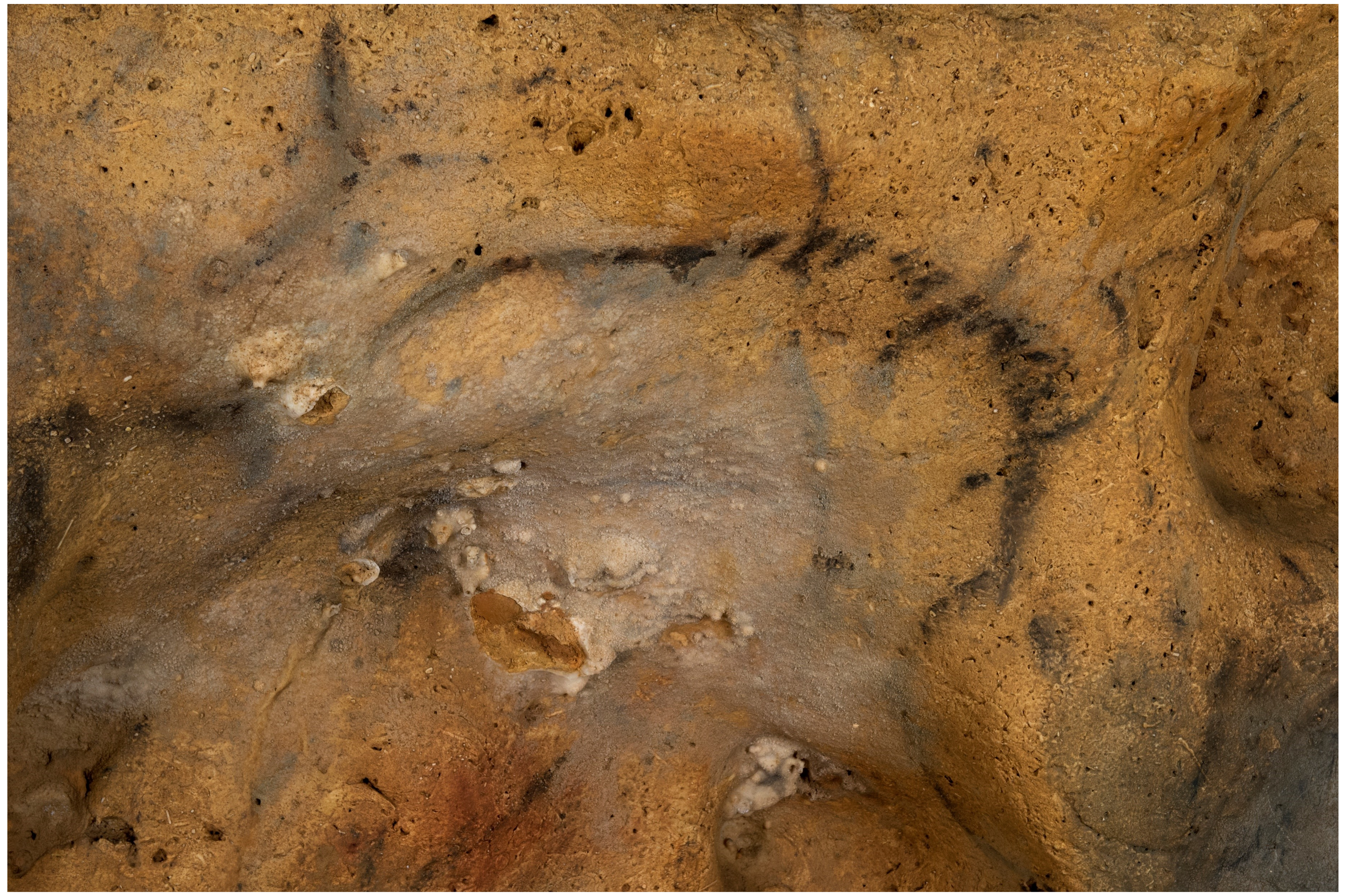

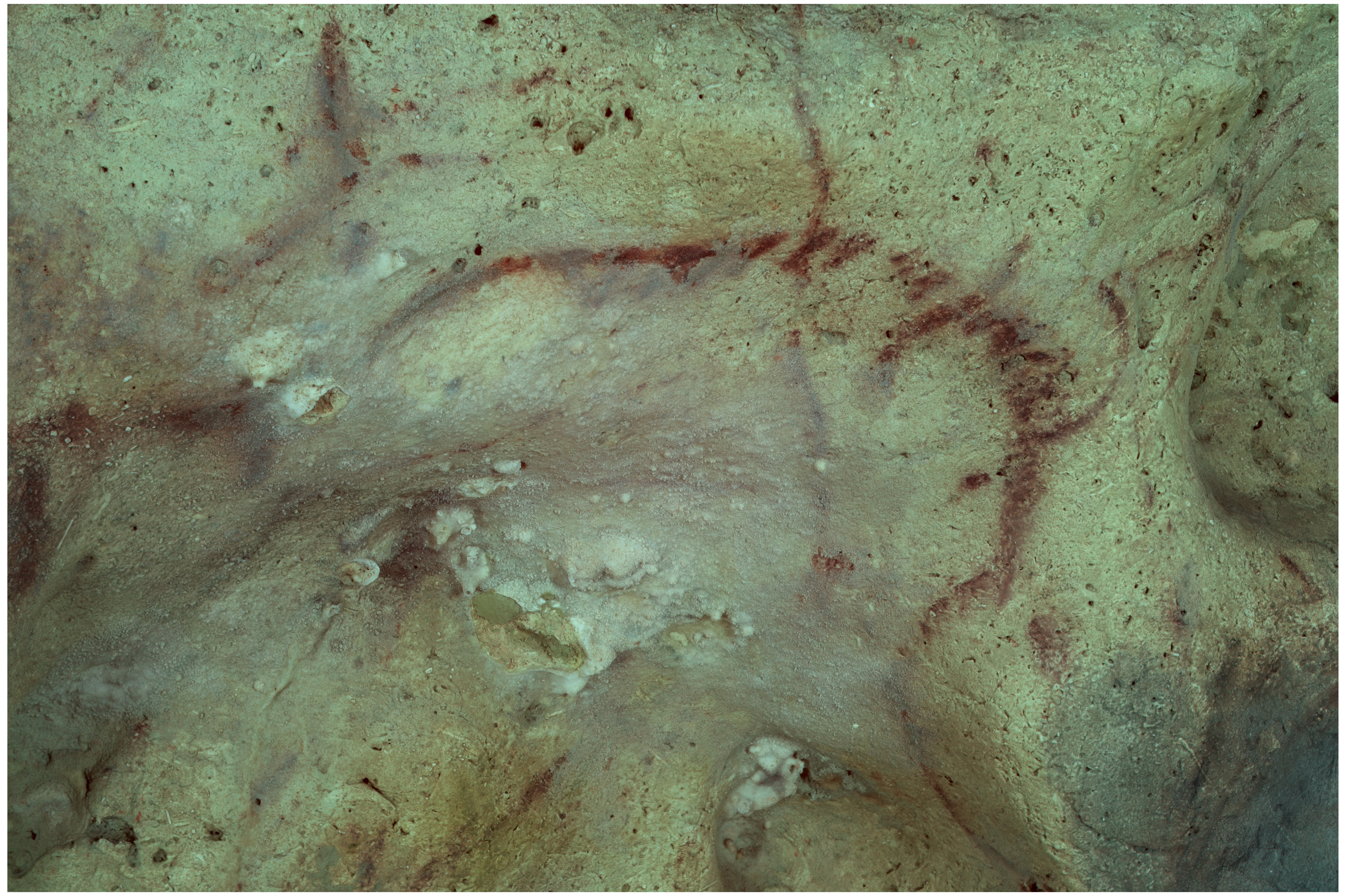

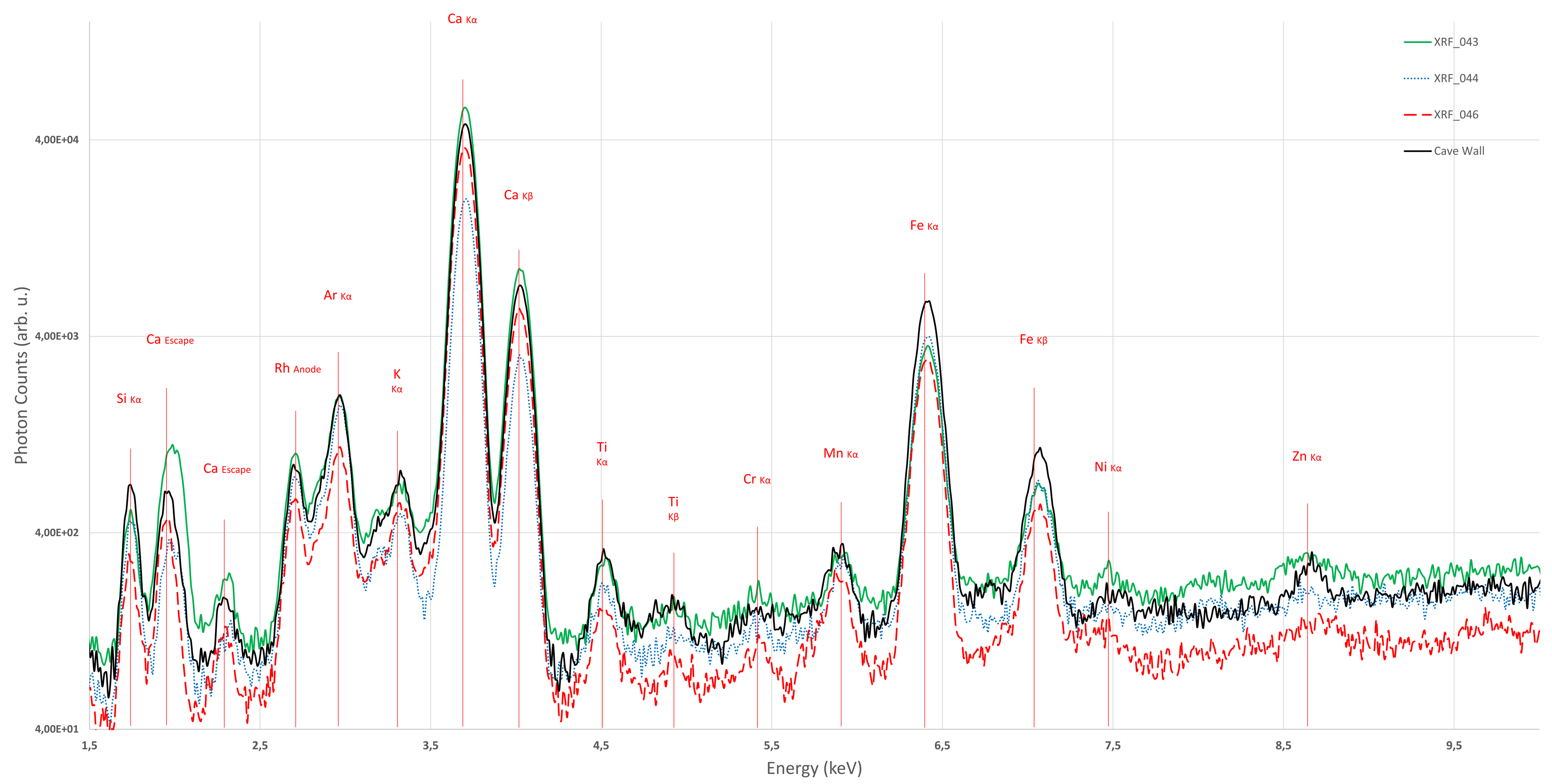

XRF spectra

Absence of Mn

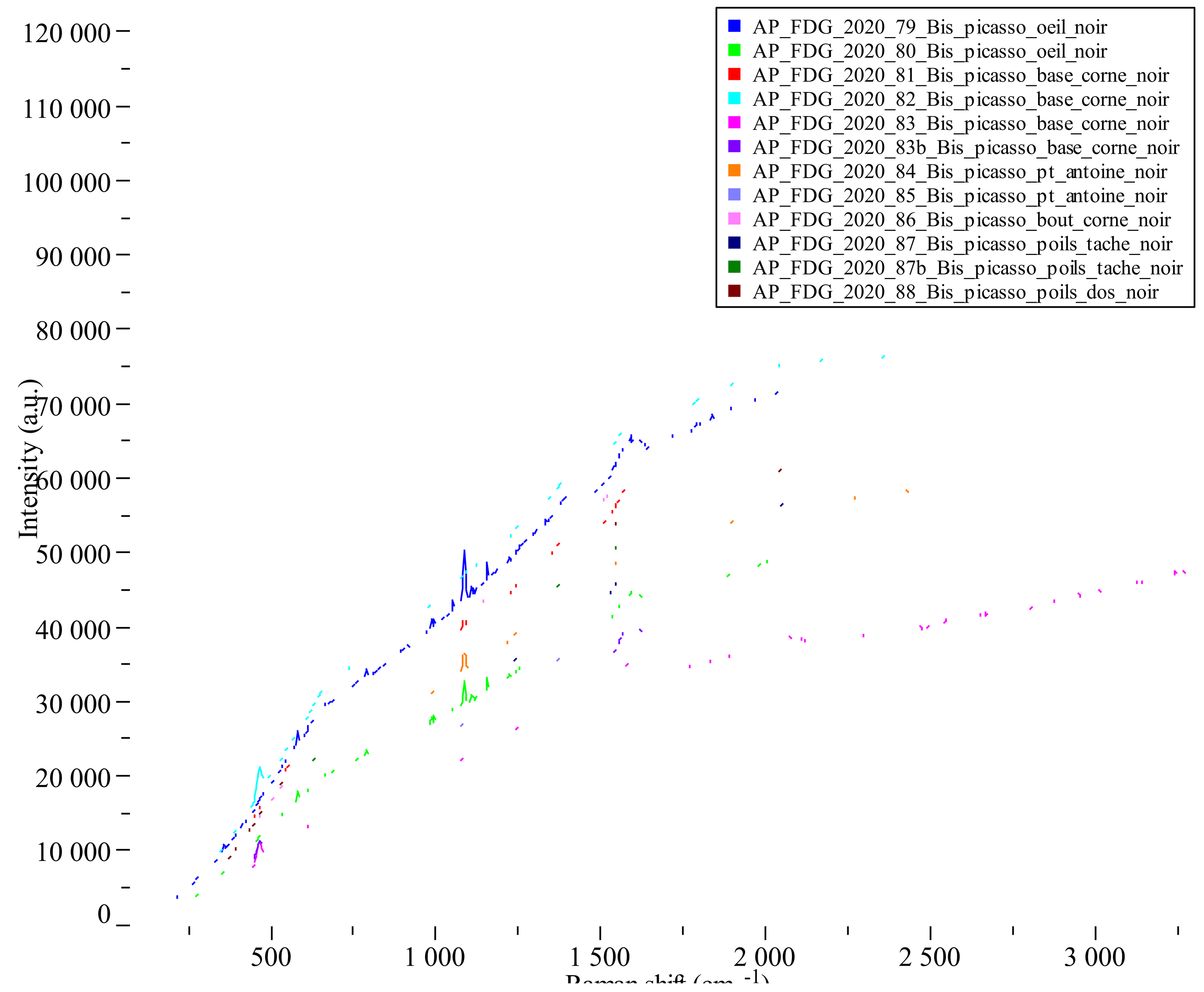

Raman spectra

charcoal
